# Global transcriptome and gene regulation network for secondary metabolite biosynthesis of tea plant (*Camellia sinensis*)

**DOI:** 10.1186/s12864-015-1773-0

**Published:** 2015-07-29

**Authors:** Chun-Fang Li, Yan Zhu, Yao Yu, Qiong-Yi Zhao, Sheng-Jun Wang, Xin-Chao Wang, Ming-Zhe Yao, Da Luo, Xuan Li, Liang Chen, Ya-Jun Yang

**Affiliations:** Key Laboratory of Tea Biology and Resources Utilization, Ministry of Agriculture, Tea Research Institute of the Chinese Academy of Agricultural Sciences, Hangzhou, 310008 China; Key Laboratory of Synthetic Biology, Institute of Plant Physiology and Ecology, Shanghai Institutes for Biological Sciences, Chinese Academy of Sciences, Shanghai, 200032 China; Suzhou Genezym Biological Technology Co, Ltd, Suzhou, 215011 China; School of Life Sciences, Sun Yat-Sen University, Guangzhou, 510275 China; Present address: The University of Queensland, Queensland Brain Institute, Brisbane St Lucia, QLD 4072 Australia

**Keywords:** Tea plant, *Camellia sinensis*, RNA-seq, Secondary metabolite, Transcription factor, Regulation network

## Abstract

**Background:**

Major secondary metabolites, including flavonoids, caffeine, and theanine, are important components of tea products and are closely related to the taste, flavor, and health benefits of tea. Secondary metabolite biosynthesis in *Camellia sinensis* is differentially regulated in different tissues during growth and development. Until now, little was known about the expression patterns of genes involved in secondary metabolic pathways or their regulatory mechanisms. This study aimed to generate expression profiles for *C. sinensis* tissues and to build a gene regulation model of the secondary metabolic pathways.

**Results:**

RNA sequencing was performed on 13 different tissue samples from various organs and developmental stages of tea plants, including buds and leaves of different ages, stems, flowers, seeds, and roots. A total of 43.7 Gbp of raw sequencing data were generated, from which 347,827 unigenes were assembled and annotated. There were 46,693, 8446, 3814, 10,206, and 4948 unigenes specifically expressed in the buds and leaves, stems, flowers, seeds, and roots, respectively. In total, 1719 unigenes were identified as being involved in the secondary metabolic pathways in *C. sinensis*, and the expression patterns of the genes involved in flavonoid, caffeine, and theanine biosynthesis were characterized, revealing the dynamic nature of their regulation during plant growth and development. The possible transcription factor regulation network for the biosynthesis of flavonoid, caffeine, and theanine was built, encompassing 339 transcription factors from 35 families, namely bHLH, MYB, and NAC, among others. Remarkably, not only did the data reveal the possible critical check points in the flavonoid, caffeine, and theanine biosynthesis pathways, but also implicated the key transcription factors and related mechanisms in the regulation of secondary metabolite biosynthesis.

**Conclusions:**

Our study generated gene expression profiles for different tissues at different developmental stages in tea plants. The gene network responsible for the regulation of the secondary metabolic pathways was analyzed. Our work elucidated the possible cross talk in gene regulation between the secondary metabolite biosynthetic pathways in *C. sinensis*. The results increase our understanding of how secondary metabolic pathways are regulated during plant development and growth cycles, and help pave the way for genetic selection and engineering for germplasm improvement.

**Electronic supplementary material:**

The online version of this article (doi:10.1186/s12864-015-1773-0) contains supplementary material, which is available to authorized users.

## Background

The production of secondary metabolites in tea plants (*Camellia sinensis* (L.) O. Kuntze) contributes to the rich flavors, clean taste, and nutrient content of tea [1, 2], one of the most popular beverages worldwide. These secondary metabolites are also known to be beneficial to human health. Animal, clinical, and epidemiological studies suggest that tea is beneficial in the prevention and treatment of chronic diseases, including cardiovascular diseases and cancer [3–5]. The secondary metabolites in tea plants include polyphenols, alkaloids, volatile oils, and others. Among them, flavonoids, caffeine, and theanine are the major constituents. Flavonoids are phenylalanine-derived, physiologically active secondary metabolites, and include flavones, flavonols, isoflavones, flavanones, flavanols, and anthocyanidins [6]. These compounds have a wide range of functions, such as antioxidant activity, ultraviolet light protection, and defense against phytopathogens. Caffeine is a purine alkaloid that has been widely used as a stimulant and an ingredient in drugs. Caffeine accumulates in seeds, buds, and young leaves, and serves as an anti-herbivory compound to protect soft tissues from predators [7]. Caffeine in seed coats is released into the soil and inhibits the germination of other seeds [8]. Theanine is a unique free amino acid and accounts for approximately 50 % of the total free amino acids in tea. This compound gives tea a unique taste known as “umami” [9]. Theanine was reported to act as an antagonist against caffeine-induced paralysis [10–13]. Additionally, it acts as a neurotransmitter in the brain and has a relaxation-inducing effect in humans [10].

Although the *C. sinensis* genome has not yet been resolved, its genes have been identified and annotated in studies using expressed sequence tags (ESTs) [14–16] and high-throughput RNA-sequencing (RNA-seq) technology [17–20]. The genes involved in many metabolic pathways have been a key focus, and efforts have been made previously to identify these genes in tea plants. Using Sanger sequencing, ESTs were generated from the tender shoots [14] and leaves of the tea plant *C. sinensis* [15, 16]. RNA-seq is particularly attractive for non-model organisms without available genomic sequences [21, 22]. More recently, RNA-seq was used to obtain full-scale transcriptomic information from mixed tissues and leaves, and the majority of the essential genes in the flavonoid, caffeine, and theanine biosynthetic pathways were characterized [17, 19]. By analyzing the transcriptome profiles from four tea plant cultivars, Wu et al. identified the critical genes that regulate catechins biosynthesis [20]. Using both RNA-seq and digital gene expression technologies, Wang et al. studied the transcription profiles of mature leaves of tea plants in response to low non-freezing temperatures and revealed the gene expression changes during cold acclimation in *C. sinensis* [18].

Secondary metabolite biosynthesis in the tea plant is regulated in different organs/tissues at different stages of development. Secondary metabolites play important roles in defense, acclimation, and communication in plants; therefore, their production is often disturbed by environmental changes and growth cycles. Until now, very little was known about the pattern of secondary metabolite biosynthesis in the different organs and tissues of tea plants or about how the expression of the genes involved in their biosynthesis is regulated during plant development and growth.

Both leaves and buds are used by the tea industry as the raw materials for tea production because of their abundance of secondary metabolites. However, the flavor of tea products varies with the conditions under which the tea plants are grown, when and how the leaves and buds are harvested, and how they are stored and processed. The chemical composition also changes with organ/tissue development. Previous researchers found that there were high levels of flavan-3-ols in young and developing leaves, low levels in stems, and extremely low levels in roots and cotyledons [23]. The content of (−)-epigallocatechin-3-*O*-gallate, which is the principal flavan-3-ol in tea leaves, decreased with the age of the leaf. Conversely, the content of (−)-epigallocatechin in young leaves was very low, but increased markedly with leaf age [24]. The amount of caffeine was higher in older leaves than in younger leaves. Theobromine, a precursor for caffeine biosynthesis, was only found in younger leaves [25]. The concentration of theanine in *C. sinensis* seedlings was higher in roots, lower in shoots, and decreased to the lowest level in cotyledons [26]. Thus, secondary metabolite biosynthesis is regulated in different tissues during the development of tea plants. It is critical to understand the patterns of secondary metabolite biosynthesis during development and how they are regulated at the transcriptional level. However, few studies are available on this important topic.

This study elucidated the global expression patterns of genes involved in metabolism, particularly secondary metabolism, and characterized their regulatory network in tea plants. We collected samples from 13 different organs and tissues at various developmental time points, including buds and leaves at various developmental stages and tissue samples of stems, flowers, seeds, and roots. After performing RNA-seq on these samples, we assembled a gene set that is more complete than previous versions and includes genes that are expressed in organs and tissues that have not been previously examined. Furthermore, we identified large sets of differentially expressed genes in each organ and tissue. In particular, the expression patterns of important genes involved in secondary metabolism were characterized, revealing the dynamic regulation of secondary metabolism during organ and tissue development. Using transcriptome data from the 13 tissues, we built co-expression networks of transcription factors and genes involved in flavonoid, caffeine, and theanine biosynthesis. Our study revealed the global gene expression profiles during organ and tissue development, and the possible regulatory network for genes important in secondary metabolite biosynthesis. This work expands the resources available for investigating the gene expression profiles of the organs and tissues of tea plant throughout the life cycle. The results not only aid our understanding of how the expression of secondary metabolite biosynthetic genes are regulated during organ and tissue development and tea plant growth, but it also represents a valuable reference for the design, formulation, and manufacturing of tea products in an industrial setting.

## Results and discussion

### Sample collection and RNA-seq of *C. sinensis* tissues

**Fig. 1:**
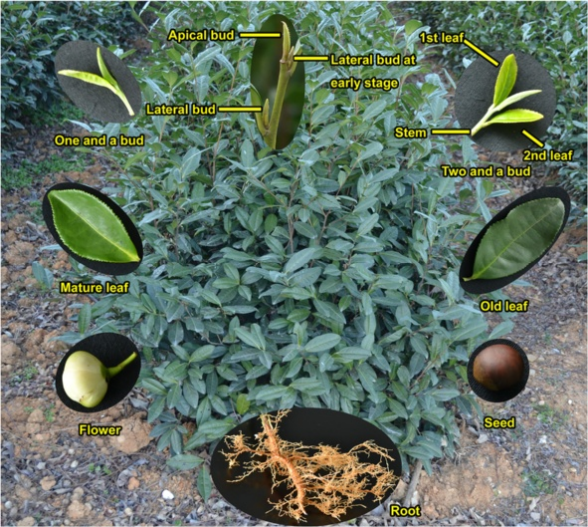
Thirteen different tissues of *C. sinensis* collected in this study. The name of each tissue is shown in yellow. The details for each tissue are described in the “Sample collection and RNA-seq of *C. sinensis* tissues” in the *Results and Discussion*

To analyze the organs/developmental tissues of *C. sinensis* systematically, a total of 13 tea plant tissues were selected for RNA-seq analysis in this study (Fig. 1), including buds and leaves at various developmental stages (apical buds, lateral buds at the early stage, lateral buds, one leaf and one bud, two leaves and one bud, first leaf, second leaf, mature leaf, and old leaf) and tissue samples from four other organs (stems, flowers, seeds, and roots). Typically, the buds and the first two or three leaves are harvested for tea production. The flavor of tea products varies with the age of the leaves and buds, as the chemical compositions change with age. Buds include apical buds and lateral buds, which are defined by their locations in the growing shoots (Fig. 1). Apical buds are unopened leaves on the top of actively growing shoots; their apical dominance can inhibit the growth of lateral buds. The lateral buds, growing between leaf axils, germinate only when the apical buds are removed or remain stunted. Lateral buds at the early stage are young buds of approximately 10 mm in length. The first leaf grows next to the apical bud, and the second leaf follows the first leaf. Green tea produced in the spring is made from the buds (apical or lateral), one leaf and one bud (one and a bud) or two leaves and one bud (two and a bud). The mature leaves germinate in the spring and are harvested in the autumn. Old leaves usually germinated in previous years, and their physiological functions are reduced, although they still have some photosynthetic capacity. The old leaves provide nutrients to tea plants and play important roles in the storage of nutrients that are required for the germination and growth of new shoots. The flowers are pollinated in the autumn, and the mature seeds are harvested in the autumn of the following year.

RNA-seq libraries were prepared from the *C. sinensis* tissues described above. Each RNA-seq library was sequenced using the Illumina HiSeq2000 platform, with a paired-end read length of 100 base pairs (bp). For each sample, sequence data ranging from 2.2 to 4.6 Gbp were generated (Table 1). A total of 437.3 million raw reads (approximately 43.7 Gbp) were obtained for all the harvested tissues from *C. sinensis*. The number of reads surpassed the total of all of the previous studies combined and represents the global landscape of gene expression resulting from the inclusion of numerous developmental stages and tissues. After the low-quality, ambiguous, and adaptor-containing sequences were removed, 375.9 million high-quality reads (32.3 Gbp) were obtained. The sequencing data provided us with an unprecedented opportunity to profile the metabolic activities in the tea plant’s critical organs and tissues and the changes that occur throughout the plant’s life cycle. Furthermore, the results provide insight into the regulatory mechanisms and the cross-talk that occur between the secondary metabolite biosynthesis pathways in *C. sinensis*.

**Table 1:** Overview of the sequencing and assembly of the *C. sinensis* transcriptome

	No. of reads	No. of bases (bp)
Apical bud	42,265,206	4,226,520,600
Lateral bud at early stage	29,347,984	2,934,798,400
Lateral bud	45,972,870	4,597,287,000
One and a bud	22,359,002	2,235,900,200
Two and a bud	33,530,490	3,353,049,000
First leaf	31,598,182	3,159,818,200
Second leaf	41,592,108	4,159,210,800
Mature leaf	31,336,700	3,133,670,000
Old leaf	33,098,022	3,309,802,200
Stem	22,211,892	2,221,189,200
Flower	31,562,386	3,156,238,600
Seed	39,633,068	3,963,306,800
Root	32,836,054	3,283,605,400
Total raw data	437,343,964	43,734,396,400
Total high-quality data	375,912,240	32,306,754,934
Average high-quality read length (bp)	86.9	
Average unigene length (bp)	791.2	
Range of unigene length (bp)	201-28,245	
Unigenes ≥ 200 bp	347,827	275,194,286
N50 (bp)	1340	

### Unigene assembly and comparative analyses for *C. sinensis* tissues

**Fig. 2:**
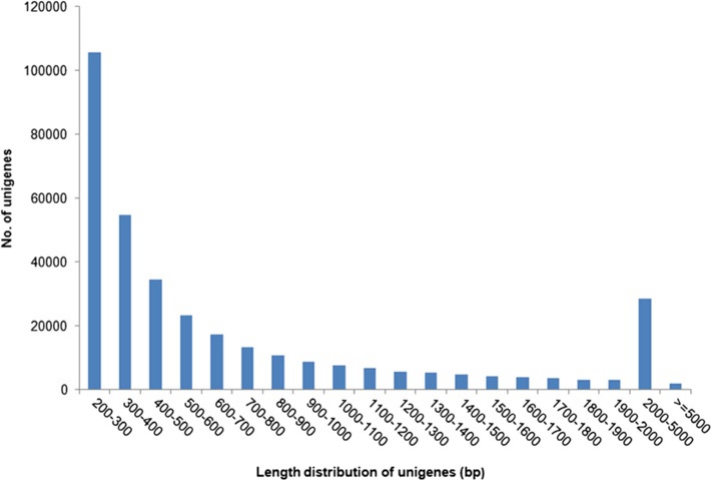
Length distributions of assembled unigenes

The high-quality reads from all tissues were combined, and the unigene assembly was performed using the program Trinity [27]. As a result, 347,827 unigenes were generated, with a total size of 275.2 Mb. The lengths of the unigenes ranged from 201 to 28,245 bp, with an average size of 791.2 bp. In total, 153,000 unigenes (43.9 %) were longer than 500 bp, and 78,870 unigenes (22.7 %) were longer than 1 kb (Fig. 2). Previously, 127,094 unigenes were assembled from mixed tissue samples of *C. sinensis*, with 17.9 % of the unigenes having a length longer than 500 bp [17].

**Fig. 3:**
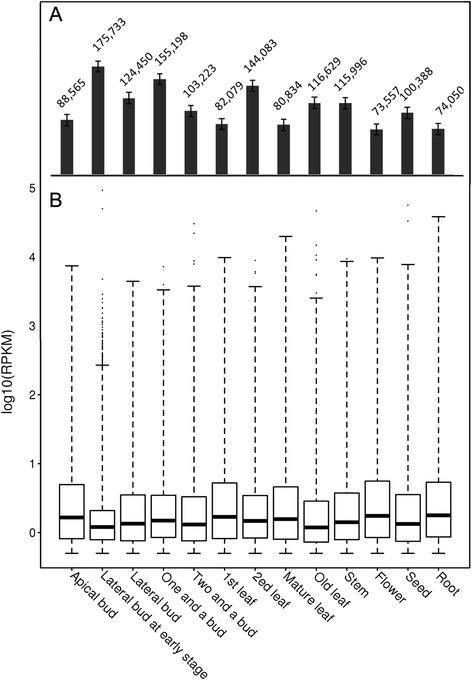
Numbers and levels of expressed unigenes from different tissues of *C. sinensis*. **a** The number of unigenes expressed in each tissue was determined, and the numbers are given over the bars. **b** Expression levels of unigenes from 13 tissues. The boxplots represent the 25–75 % intervals of unigene expression for each tissue

Reads from the 13 different tissues were mapped to the assembled unigenes using Bowtie2 [28]. The levels of the unigenes were measured in each sample by the RPKM (reads per kilobase per million reads) values, with an RPKM ≥ 0.5 being considered expressed. The number of genes expressed and the distribution of their expression levels are shown in Fig. 3a and b. In general, a greater number of expressed genes were detected in the bud and leaf tissues than in the flower and root tissues (Fig. 3a); however, a similar distribution of gene expression was observed in all of the tissues (Fig. 3b).

**Fig. 4:**
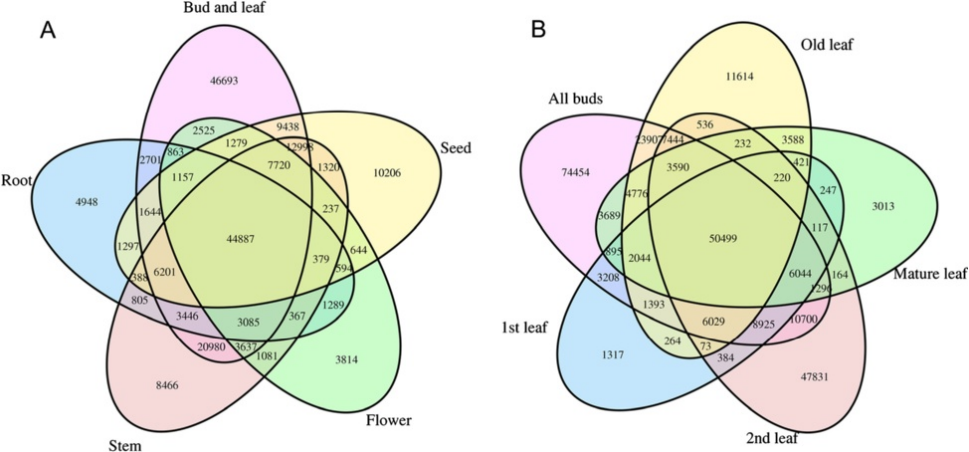
Venn diagram of co-expressed and uniquely expressed unigenes from different tissues of *C. sinensis*. **a** Statistics regarding the co-expressed and uniquely expressed unigenes in all of the tissues studied. The “bud and leaf” group comprised the samples “one and a bud” and “two and a bud”, and the other labels represent the indicated tissues. **b** Statistics regarding the co-expressed and uniquely expressed unigenes in the buds and leaves. The “All buds” sample comprised the samples “apical bud”, “lateral bud at early stage”, and “lateral bud”, and the other labels represent the indicated tissues

We next asked whether the differences in gene expression occur between different organs and tissues or between different developmental stages. We first compared tea plant buds and leaves, stems, flowers, seeds, and roots (Fig. 4a). For the “bud and leaf” type, we combined the data for “one and a bud” and “two and a bud”. The five tissue types shared 44,887 unigenes, with the number of tissue-specific unigenes ranging from 46,693 in “bud and leaf” to 3814 in flowers. The “bud and leaf” category appears to contain more unigenes than the other tissues, most likely because it includes two tissues (bud and leaf) and multiple developmental stages, resulting in the expression of more genes because of the complex development and differentiation processes that occur in buds and leaves. To better understand the gene expression changes that take place during the development of buds and leaves, we examined the gene expression patterns in greater detail. The comparison included five tissue groups, “all buds” (containing apical buds, lateral buds at early stage and lateral buds), first leaf, second leaf, mature leaf, and old leaf. All of these bud and leaf tissues shared 50,499 unigenes, which likely correspond to genes that provide essential functions in buds and leaves (Fig. 4b). As expected, the buds expressed the largest number of tissue-specific unigenes (74,454), again most likely as a result of the merging of the bud tissues of three developmental stages.

### Functional annotation of the transcriptomes of 13 *C. sinensis* tissues

All of the *C. sinensis* unigenes were annotated based on sequence similarity using five public databases, including the National Center for Biotechnology Information (NCBI) non-redundant protein database (Nr) [29], the Arabidopsis Information Resource (TAIR) [30], the Swiss-Prot protein database (Swiss-Prot) [31], the Translated EMBL Nucleotide Sequence database (TrEMBL), [32] and the Conserved Domain Database (CDD) [33] (Table 2). In total, 176,356 (50.7 %) *C. sinensis* unigenes were annotated, while the remaining 171,471 (49.3 %) had no significant matches to any sequences in the public databases. This result is in line with a previous work in which the authors found 72,006 (56.7 %) unigenes that had no resemblance to genes from sources other than tea plants [17]. Our work and the work of others have revealed numerous new genes specific to *C. sinensis* with unknown functions, which will be the subject of future studies.

**Table 2:** Summary of unigene annotations

Database	Total unigenes	Annotated unigenes	Percent (%)	Non-annotated unigenes	Percent (%)
Nr	347,827	140,989	40.5	206,838	59.5
Swiss-Prot	347,827	104,457	30.0	243,370	70.0
CDD	347,827	139,501	40.1	208,326	59.9
TrEMBL	347,827	155,771	44.8	192,056	55.2
TAIR	347,827	73,048	21.0	274,779	79.0
Total	347,827	176,356	50.7	171,471	49.3

**Fig. 5:**
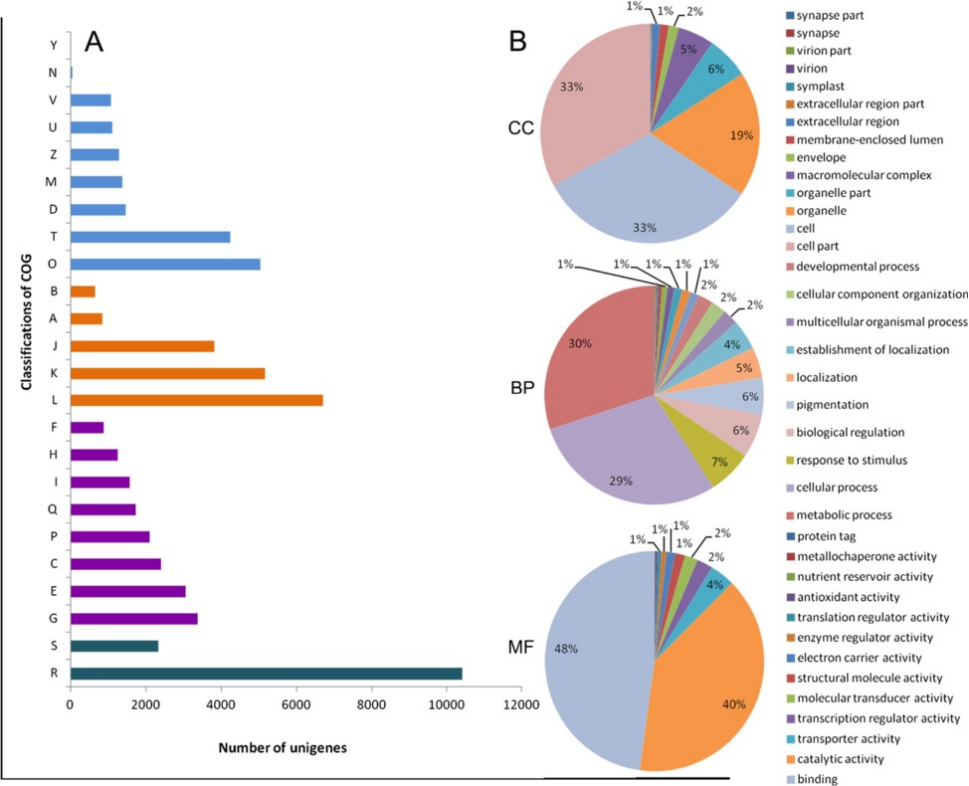
Functional classifications of unigenes from *C. sinensis*. **a** COG (cluster of orthologous groups) classifications of the unigenes. A: RNA processing and modification; B: Chromatin structure and dynamics; C: Energy production and conversion; D: Cell cycle control, cell division, chromosome partitioning; E: Amino acid transport and metabolism; F: Nucleotide transport and metabolism; G: Carbohydrate transport and metabolism; H: Coenzyme transport and metabolism; I: Lipid transport and metabolism; J: Translation, ribosomal structure and biogenesis; K: Transcription; L: Replication, recombination and repair; M: Cell wall/membrane/envelope biogenesis; N: Cell motility; O: Posttranslational modification, protein turnover, chaperones; P: Inorganic ion transport and metabolism; Q: Secondary metabolites biosynthesis, transport and catabolism; R: General function prediction only; S: Function unknown; T: Signal transduction mechanisms; U: Intracellular trafficking, secretion, and vesicular transport; V: Defense mechanisms; Y: Nuclear structure; Z: Cytoskeleton; **b** Gene ontology (GO) classifications of unigenes in 13 tissues. The three pie charts show the major categories of GO terms, which are Cellular Component (CC), Biological Process (BP), and Molecular Function (MF). Each major category was further divided into many sub-categories whose proportions are shown underneath the labels

Clusters of orthologous groups (COGs) consist of protein sequences encoded in 21 prokaryotic and eukaryotic genomes, which were built based on classifications according to phylogenetic relationships [34]. Each COG contains protein homologs from two or more lineages, related to conserved domains of ancient origin. In our study, a total of 56,751 unigenes were assigned to 24 COG clusters (Fig. 5a). Some unigenes were annotated with multiple COG functions; therefore, altogether 61,558 functional annotations were produced. The five largest categories included: 1) general functions (18.3 %) associated with basic physiological and metabolic functions; 2) replication, recombination, and repair (11.8 %); 3) transcription (9.1 %); 4) post-translational modification, protein turnover, and chaperones (8.8 %); and 5) signal transduction mechanisms (7.4 %). Unigenes involved in secondary metabolism (secondary metabolite biosynthesis, transport, and catabolism) represented approximately 3.0 % (1719 unigenes) of all COG-annotated unigenes. Among them, 262 unigenes encode SAM-dependent methyltransferases, and 54 unigenes encode naringenin-chalcone synthase. SAM-dependent methyltransferases play key roles in three methylation steps in caffeine biosynthesis, and catalyze the initial step and the last two steps of purine modification. Naringenin-chalcone synthase catalyzes the initial step of flavonoid biosynthesis.

Unigenes from *C. sinensis* were classified using Gene Ontology (GO), which provides a canonical vocabulary of functional terms for genes across all species [35]. All unigenes in our study were assigned GO terms based on Blastx searches against the Nr dataset. A total of 86,102 unigenes were assigned GO terms, which could be summarized into three main categories (cellular component, biological process, and molecular function) (Fig. 5b). In the cellular component (CC) category, the cell, cell part, organelle, organelle part, and macromolecular complex corresponded to 33, 33, 19, 6, and 5 % of the unigenes, respectively (Fig. 5b). The major subgroups of biological processes (BP) included metabolic process (30 %), cellular process (29 %), response to stimulus (7 %), biological regulation (6 %), and pigmentation (6 %) (Fig. 5b). The best-represented groups of molecular function (MF) were binding activity, catalytic activity, and transporter activity, which account for 92 % of the total unigenes mapped to MF (Fig. 5b).

GO terms for all of the unigenes and tissue-specific unigenes in each sample were enriched according to a hypergeometric test. For each tissue, the enriched categories are listed in Additional file 1: Table S1. The cell, cell part, and cellular component were the most dominant subcategories in the apical bud, second leaf, stem, and flower. Cellular nitrogen compound metabolic process was the predominant subcategory in the lateral buds at the early stage, suggesting that the chemical reactions related to nitrogenous compounds are active in this tissue. DNA metabolic process was the enriched category in the lateral buds at the early stage and in old leaves, indicating that DNA metabolism was an active biological process in these tissues. To investigate the tissue functions, the tissue-specific unigenes were enriched for the GO categories of each tissue (Additional file 1: Table S2). Among the tissues, the roots were distinguished from the other tissues based on the enrichment in the GO categories of detection of external stimulus, heme binding, and iron ion binding; the other tissues commonly had GO categories of DNA metabolic process, macromolecule metabolic process, and RNA binding. The GO category “transferase activity, transferring phosphorus-containing groups” was enriched in all buds, bud and leaf, mature leaf, old leaf and seed, which indicated that the specific unigenes in these tissues were actively involved in catalyzing the transfer of a phosphorus-containing group. The GO category purine nucleoside binding was present in second leaves and old leaf tissues. Purine nucleoside biosynthesis is important in caffeine biosynthesis, and this observation implied that the genes involved in the caffeine biosynthesis pathway were expressed in the second leaves.

### Analysis of important secondary metabolite biosynthetic pathways in different *C. sinensis* tissues

Flavonoids, caffeine, and theanine are the three major secondary metabolites in *C. sinensis*, and they are important contributors to the flavor of tea. We focused our analyses on the biosynthetic pathways of these metabolites and the differential expressions of the related genes in the 13 *C. sinensis* tissues. Based on the KEGG database, a total of 206 unigenes were annotated and found to be associated with the biosynthetic pathways of the three metabolites.

### Flavonoid biosynthesis

Flavonoids are a group of plant polyphenol secondary metabolites that includes flavones, flavonols, isoflavones, flavanones, flavanols, and anthocyanidins. The flavan-3-ols, or catechins, are the most prominent flavonoid compounds in leaves [36, 37]. These compounds contribute to many of the features that make tea a highly valuable component of the human diet.

**Fig. 6:**
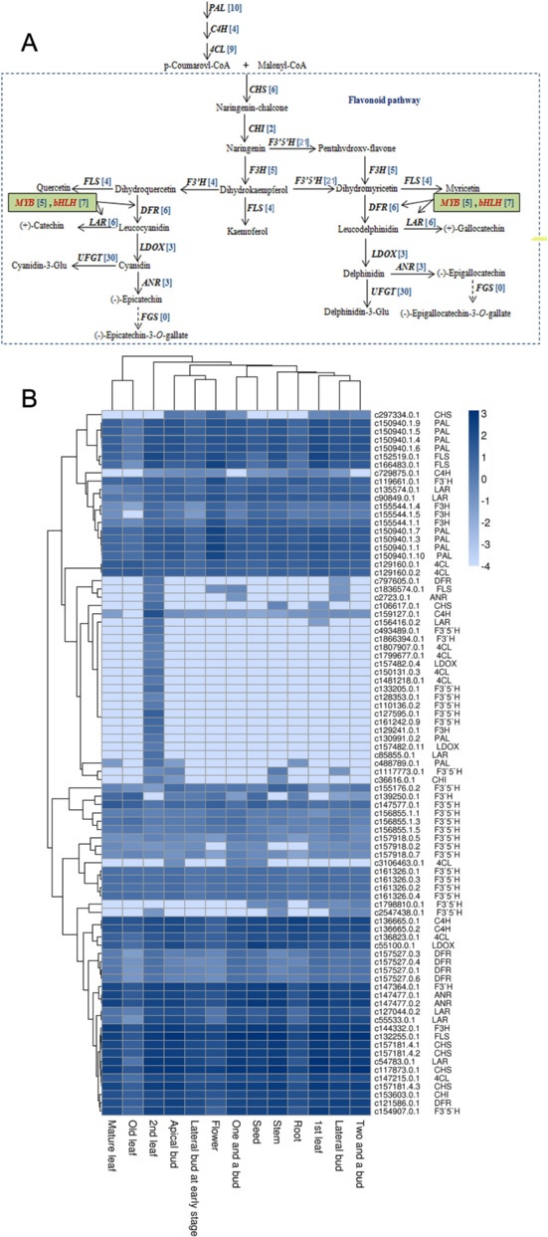
Putative flavonoid biosynthetic pathway in *C. sinensis*. **a** The flavonoid biosynthetic pathway. The blue numbers in the brackets following each gene name indicate the number of corresponding unigenes. *PAL*, phenylalanine ammonia-lyase; *C4H*, cinnamic acid 4-hydroxylase; *4CL*, 4-coumarate-CoA ligase; *CHS*, chalcone synthase; *CHI*, chalcone isomerase; *F3H*, flavanone 3-hydroxylase; *F3′,5′H*, flavonoid 3′,5′-hydroxylase; *F3′H*, flavonoid 3′-hydroxylase; *FLS*, flavonol synthase; *DFR*, dihydroflavonol 4-reductase; *ANS*, anthocyanidin synthase; *ANR*, anthocyanidin reductase; *LAR*, leucocyanidin reductase; *FGS*, flavan-3-ol gallate synthase; *LDOX*, leucoanthocyanidin oxidase; *UFGT*, UDP-glucose: flavonoid 3-*O*-glucosyl transferase. **b** Heat map of the expression levels of the flavonoid biosynthetic unigenes in different tissues. The tissues are listed horizontally, and the unigenes are listed on the vertical line. The annotations are displayed on the right side. The scale represents the logarithms of the RPKM (reads per kilobase per million reads) values of the unigenes. The unigenes were clustered using the “Pearson correlation”, whereas the tissues were clustered using the “Maximum distance”

The central pathways for flavonoid biosynthesis are highly conserved and well characterized [38–40]. Flavonoids are synthesized in the general phenylpropanoid pathway. In the flavonoid pathway, chalcone synthase (CHS) catalyzes the first step in the biosynthesis of flavonoids. Flavanone 3-hydroxylase (F3H) catalyzes the formation of dihydroflavonols from flavanones (Fig. 6a). We found five unigenes annotated as *F3H* according to the KEGG pathway. Among them, three unigenes (c155544.0.1, c155544.0.4, and c144332.0.1) were globally expressed in all of the tissues (Fig. 6b). Of the other two unigenes, one (c155544.0.5) was expressed in most of the tissues, but not in old leaves, and the other (c129241.0.1) was only expressed in second leaves. The three *F3H* unigenes (c155544.0.1, c155544.0.4, and c155544.0.5) may be transcribed from the same gene by alternative splicing. The dihydroflavonols serve as intermediates for the biosynthesis of flavonols, flavan-3-ols, and anthocyanidins [38]. In tea, the flavonoid pathway has been implicated in the biosynthesis of catechins [24]. Leucoanthocyanidins are the direct precursors of flavan-3-ols (e.g., catechin and gallocatechin) produced by leucoanthocyanidin reductase (*LAR*). There were six *LAR* unigenes (c135574.0.1, c90849.0.1, c156416.0.2, c85855.0.1, c127044.0.2, c55533.0.1) in our database. One unigene (c85855.0.1) was specifically expressed in second leaves, and another unigene (c156416.0.2) was expressed in both first and second leaves. The other *LAR* unigenes were globally expressed in all tissues (Fig. 6b).

The formation of epi-flavan-3-ols (e.g., epicatechin, epigallocatechin) can be achieved through a two-step reaction on leucoanthocyanidin by leucoanthocyanidin oxidase (LDOX) and anthocyanidin reductase (ANR) (Fig. 6a). There were three *LDOX* and three *ANR* unigenes from tea plants. Among them, one *LDOX* unigene (c55100.0.1) and two *ANR* unigenes (c147477.0.1 and c147477.0.2) were highly expressed in seeds and marginally expressed in leaves and flowers (Fig. 6b), which is consistent with the expression patterns of their homologs from *Theobroma cacao* [41].

The most abundant flavan-3-ols were gallic acid esters of epigallocatechin and epicatechin, namely, epigallocatechin-3-O-gallate and epicatechin-3-O-gallate. Flavanol-3-O-gallate synthase (FGS) catalyzes the conversion from epi(gallo)catechin to epi(gallo)catechin-3-O-gallate. However, FGS has not yet been identified. The last common step for the production of anthocyanins involves the glycosylation of cyanidin and delphinidin by the enzyme UDP-glucose: flavonoid 3-*O*-glucosyl transferase (UFGT) (Fig. 6a). Thirty candidate unigenes were identified by the Swiss-Prot database. However, further study is required to identify the unigenes that participate in anthocyanin biosynthesis in *C. sinensis*.

### Caffeine biosynthesis

**Fig. 7:**
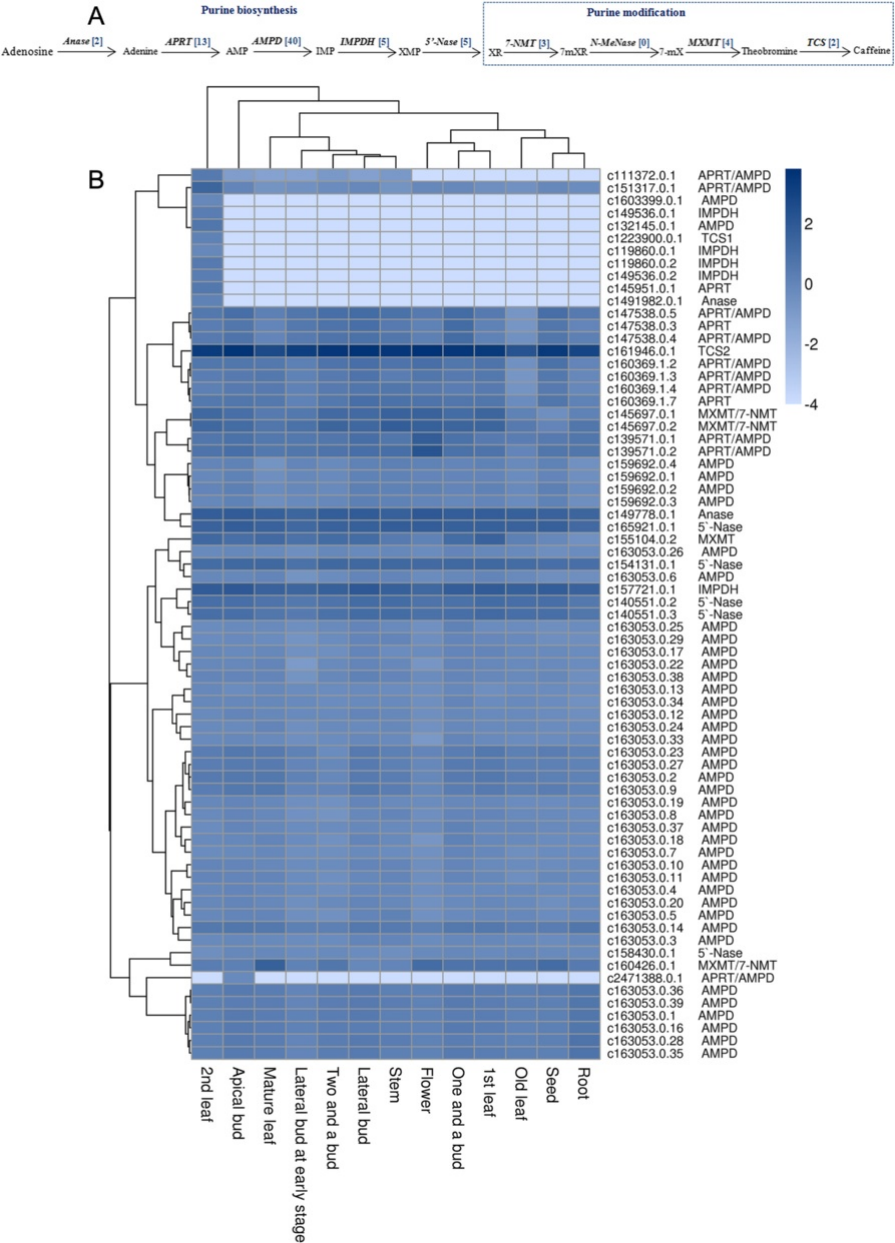
Putative caffeine biosynthetic pathway in *C. sinensis*. **a** The caffeine biosynthetic pathway. The blue number in the bracket following each gene name indicates the number of unigenes. *Anase*, adenosine nucleosidase; *APRT*, adenine phosphoribosyltransferase; *AMPD*, AMP deaminase; *IMPDH*, IMP dehydrogenase; *5′-Nase*, 5′-nucleotidase; *7-NMT*, 7-methylxanthosine synthase; *N-MeNase*, N-methylnucleotidase; *MXMT*, theobromine synthase; *TCS*, tea caffeine synthase. **b** Expression levels of candidate caffeine biosynthetic unigenes expressed in each tissue. The tissues are listed horizontally, and the unigenes are listed vertically. The gene names corresponding to the genes that were found in public databases are listed on the right. All of the RPKM (reads per kilobase per million reads) values of the unigenes are shown as logarithms. The “Pearson correlation” was used when genes in rows were clustered, and the “Maximum distance” was used when tissues in columns were clustered

Caffeine (1,3,7-trimethylxanthine) is a purine alkaloid, and the leaves of tea plants usually contain 2–5 % caffeine (dry weight) [42, 43]. In tea plants, the highest level of caffeine biosynthesis occurs in young leaves [25, 44] and fruits [45], and it decreases markedly with tissue age. The caffeine biosynthetic pathway comprises purine biosynthesis and purine modification steps. The xanthene skeleton of caffeine is derived from purine nucleotides and is catalyzed by five enzymes: adenosine nucleosidase (*Anase*), adenine phosphoribosyltransferase (APRT), AMP deaminase (AMPD), IMP dehydrogenase (IMPDH), and 5′-nucleotidase (5′-Nase) (Fig. 7a). There were two *Anase* candidate unigenes in our database, and one (c1491982.0.1) was specifically expressed in second leaves, while the other (c149778.0.1) was globally expressed in all tissues at a high level (Fig. 7b). AMPD is critical in the purine nucleotide pathway, which coverts adenosine 5′-monophosphate (AMP) into inosine 5′-monophosphate (IMP) and ammonia. AMPD forms a deaminase complex that is encoded by a multi-gene family [46]. We found 40 candidate *AMPD* unigenes in *C. sinensis*, and most of them were globally expressed in all 13 tissues. Only two *AMPD* unigenes (c1603399.0.1 and c132145.0.1) were specifically expressed in second leaves. There were five candidate *IMPDH* unigenes, and four unigenes (c149536.0.1, c119860.0.1, c119860.0.2, and c149536.0.2) were expressed specifically in second leaves at a low level, while the other (c157721.0.1) was expressed globally in all tissues.

The purine modification steps include three methylations and one nucleosidase reaction (Fig. 7a), involving 7-methylxanthosine synthase (7-NMT), N-methylnucleotidase (N-MeNase), theobromine synthase (MXMT), and tea caffeine synthase (TCS). The methylation of xanthosine is initiated by 7-NMT, and we found three *7-NMT* unigenes (c145697.0.1, c145697.0.2, and c160426.0.1). All of these unigenes were highly homologous to *MXMT,* as shown by the high sequence similarity between *7-NMT* and *MXMT. N-MeNase* has not been cloned previously and thus could not be identified in our database. TCS, the SAM-dependent methyltransferase involved in the last two steps of caffeine biosynthesis, was originally purified from the young leaves of the tea plants [47]. We found two unigenes encoding TCS in our database. One (c1223900.0.1) was expressed specifically in the second leaves, and the other (c161946.0.1) was expressed globally in all tissues and at higher levels in buds, young leaves, and stems (Fig. 7b). This result is consistent with a previous study, which showed that caffeine was synthesized at a high rate in young leaves and that its synthesis decreased with the age of the leaves [25, 44].

### Theanine biosynthesis

**Fig. 8:**
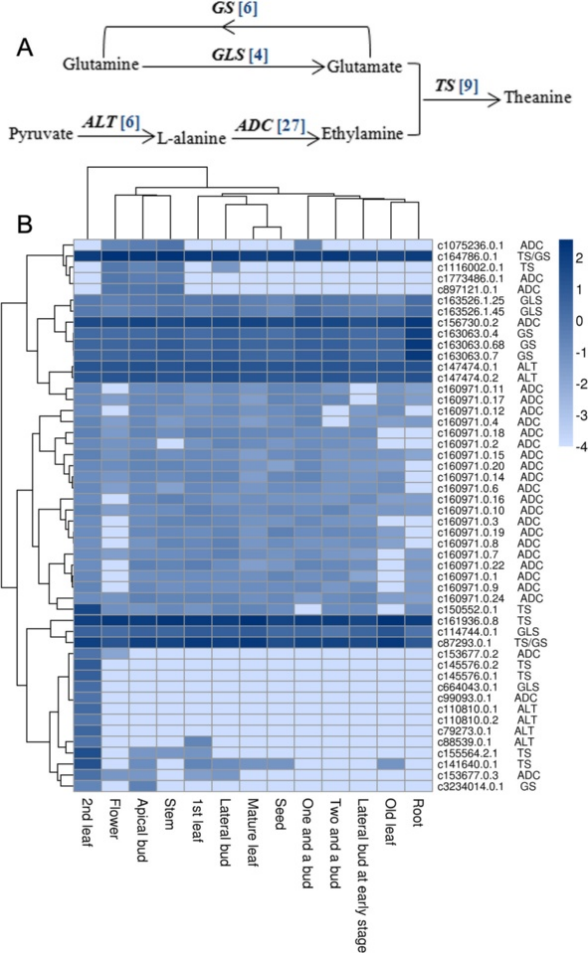
Putative theanine biosynthetic pathway in *C. sinensis*. **a** The theanine biosynthetic pathway. The blue numbers in the brackets following each gene name indicate the numbers of unigenes. *GS*, glutamine synthetase; *GLS*, glutaminase; *ALT*, alanine aminotransferase; *ADC*, arginine decarboxylase; *TS*, theanine synthetase. **b** Expression levels of candidate theanine biosynthetic unigenes expressed in different tissues. The tissues are listed horizontally, and the unigenes are listed vertically. The corresponding gene names from public transcript databases are listed on the right. All of the RPKM (reads per kilobase per million reads) values of the unigenes are shown as logarithms. Pearson correlation was used when genes in rows were clustered, and the maximum distance was used when tissues in columns were clustered

Theanine is an abundant non-protein-derived amino acid in the tea plant. Many of these amino acids are involved in producing the distinctive aroma and taste of tea, and theanine has been linked with the umami flavor of tea [9]. Theanine biosynthesis starts from glutamine and pyruvate, and involves glutamine synthetase (GS), glutaminase (GLS), alanine aminotransferase (ALT), arginine decarboxylase (ADC), and theanine synthetase (TS) (Fig. 8a). Theanine biosynthesis occurs in the buds, leaves, and roots of tea plants [26]. Six *GS* unigenes were identified, five of which were globally expressed in all tested tissues. The other one (c3234014.0.1) was specific to the apical buds and the second leaves (Fig. 8b). ALT converts pyruvate to alanine, and six *ALT* unigenes were found, each having a unique expression profile. Two *ALT* unigenes (c147474.0.1 and c147474.0.2) were highly expressed in all 13 tissues. Three other *ALT* unigenes (c110810.0.1, c110810.0.2, and c79273.0.1) were expressed only in the second leaves, and one (c88539.0.1) was expressed in the first and the second leaves. The substrate ethylamine is derived from the decarboxylation of alanine by ADC [48]. In this study, 27 putative *ADC* unigenes were identified, of which 20 had the same expression profiles among the different tissues. These unigenes may represent products of the same gene generated through alternative splicing. TS is unique in tea plants, and nine candidate *TS* unigenes were identified in our database. In addition, two of them (c16478.0.1 and c87293.0.1) were homologous to *GS*. While three *TS* unigenes (c16478.0.1, c161936.0.1, and c87293.0.1) were expressed in all the examined tissues, the other six unigenes had distinct expression patterns. Among them, two *TS* unigenes (c145576.0.1 and c145576.0.2) were expressed in the second leaves, and one (c150552.0.1) was found in most tissues, with the exception of one and a bud and old leaves. The other three unigenes (c1116002.0.1, c155564.2.1, and c141640.0.1) had specific expression patterns in different tissues (Fig. 8b). Thus, we identified and profiled a more complete set of genes that is essential in the theanine biosynthetic pathway, including the *TS*s, which were missed in previous studies [17].

**Fig. 9:**
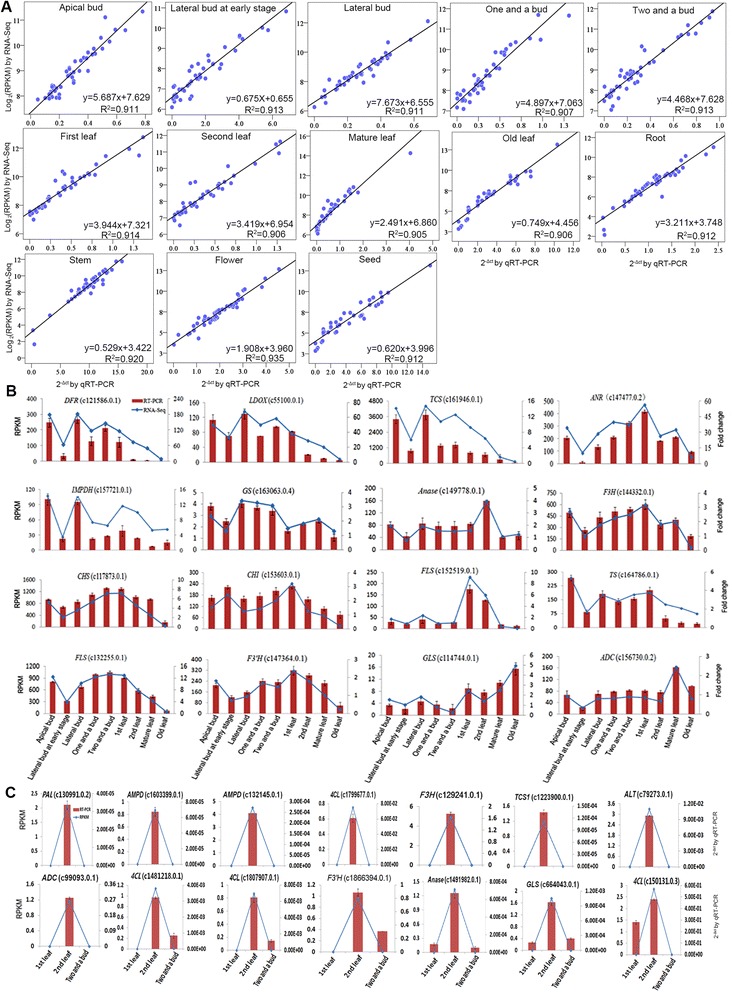
Verification of the relative expression levels of genes by quantitative RT-PCR (qRT-PCR). **a** Correlation of the expression levels of 45 randomly selected genes measured by qRT-PCR and RNA-seq. **b** Expression patterns of 16 unigenes involved in the flavonoid, caffeine, and theanine biosynthetic pathways by qRT-PCR (*Red bar*) and RNA-seq (*Blue line*). *DFR*, dihydroflavonol 4-reductase; *LDOX*, leucoanthocyanidin oxidase; *TCS*, tea caffeine synthase; *ANR*, anthocyanidin reductase; *IMPDH*, IMP dehydrogenase; *GS,* glutamine synthetase; *Anase*, adenosine nucleosidase; *F3H*, flavanone 3-hydroxylase; *CHS*, chalcone synthase; *CHI*, chalcone isomerase; *FLS*, flavonol synthase; *TS,* theanine synthetase; *F3′H*, flavonoid 3′-hydroxylase; *GLS*, glutaminase; *ADC*, arginine decarboxylase. **c** Expression patterns of 14 unigenes that were specifically expressed in second leaf according to the RNA-seq results and in first leaf, second leaf, and two and a bud according to the qRT-PCR (*Red bar*) and RNA-seq (*Blue line*) results

To validate the unigene expression changes in different tissues after quantification using the RPKM values, we randomly selected 45 unigenes and analyzed their expression levels in 13 different tissues by quantitative RT-PCR (qRT-PCR). The correlation between the RNA-seq data and the qRT-PCR results was determined by Pearson’s correlation coefficient. As a result, high correlations (R^2^ > 0.9) were found between RNA-seq and qRT-PCR (Fig. 9a), indicating that the measured changes in gene expression detected by RNA-seq reflected the actual transcriptome differences between the different tea plant tissues. In addition, we selected 16 unigenes encoding key enzymes involved in the flavonoid, theanine, and caffeine biosynthetic pathways and analyzed their expression levels in different tissues by qRT-PCR. The expression levels of most of the unigenes were consistent with the RNA-seq results (Fig. 9b). The minor discrepancy between RNA-seq and qRT-PCR for some genes (e.g., c161946.0.1) could be caused by the influence of homologous genes or the different sensitivities of RNA-seq and qRT-PCR. Finally, we selected 14 unigenes that were uniquely expressed in the second leaf, as indicated by the RNA-seq results (Figs. 6b, 7b, and 8b), and analyzed their expression levels by qRT-PCR (Fig. 9c). All of these genes exhibited a higher expression level in the second leaf tissue and had lower or no expression in the first leaf and two and a bud tissues. Among these unigenes, eight (c130991.0.2, c1799677.0.1, c129241.0.1, c1603399.0.1, c132145.0.1, c1223900.0.1, c79273.0.1, and c99093.0.1) were specifically expressed in the second leaf, which was consistent with the results of RNA-seq (Figs. 6b, 7b, and 8b). Three unigenes (c1481218.0.1, c1807907.0.1, and c1866394.0.1) presented higher expression in the second leaf, lower expression in two, and a bud and no expression in the first leaf. Two unigenes (c1491982.0.1 and c664043.0.1) were expressed in all three tissues, and the expression levels were higher in the second leaf than in the other tissues. Only one unigene (c150131.0.3) was more highly expressed in the second leaf, with lower expression in the first leaf and no expression in the two and a bud. These results showed that the expression trends detected by RNA-seq and qRT-PCR were consistent; both methods revealed that the unigenes presented higher expression in the second leaf than the other tissues. The unigenes specifically expressed in the second leaf identified by RNA-seq were also shown by qRT-PCR to be expressed in the first leaf or two and a bud tissue, which may be because these unigenes were expressed at a low level (their RPKM values ranged from 0.8 to 5). Some of the unigenes were expressed at levels that were too low to be detected by RNA-seq in the first leaf and two and a bud tissues. These results also indicated that qRT-PCR was more sensitive than RNA-seq.

### Dynamic expression of unigenes involved in secondary metabolite biosynthesis in different tissues and at different developmental stages

Extensive secondary metabolite biosynthesis is known to occur in plants. These metabolites are predominantly synthesized within specific tissues at specific developmental stages. However, in the current study, we observed that important secondary metabolite biosynthesis genes were widely expressed in mature tea plants. These genes were highly expressed in actively growing young leaves, and their expression levels decreased with senescence.

**Fig. 10:**
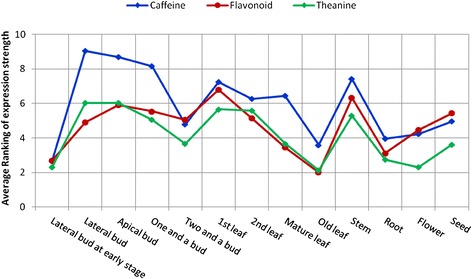
Changes in the relative expression levels of genes in the flavonoid, caffeine*,* and theanine biosynthetic pathways in 13 tissues from *C. sinensis*. The expression levels (in RPKM; reads per kilobase per million reads) of each unigene in the flavonoid, caffeine, and theanine biosynthetic pathways were ranked among the 13 tissues. An average ranking of all of the unigenes for a tissue was obtained by dividing the sum of all unigene rankings by the number of unigenes in the pathway. For a tissue, the average ranking of all of the unigenes from a biosynthetic pathway represents the relative expression strength of that pathway

To define the pattern of the overall expression changes in each secondary metabolite biosynthetic pathway, we ranked each tissue (among the 13 tissues examined) according to the expression of each gene in the secondary metabolic pathways. Then, the rankings of all genes within a particular pathway were averaged for each tissue to score the overall “strength” of the pathway in the tissue. When we plotted the average rankings of the flavonoid, caffeine, and theanine biosynthetic pathways, we found that they generally followed the same pattern of regulation as the gene expression (Fig. 10). These genes peaked first in the apical bud and lateral bud stages, and remained elevated in the first and second leaves. Their overall expression dropped in the mature and old leaves. Therefore, the unigenes of these three secondary metabolic pathways presented increasing expression levels from the young bud to the actively growing leaf stages, and the expression of these unigenes then decreased in the mature leaf and senescence stages. With the exception of the stems, in the other organs and tissues, i.e., the flowers, seeds, and roots, the genes of the flavonoid, caffeine, and theanine biosynthetic pathways were expressed at relatively lower levels. In the stems, the overall levels were comparable to those of the leaves. These results suggested that the expression of unigenes that participate in flavonoid, caffeine, and theanine biosynthesis is dynamically regulated in the tissues of *C. sinensis* during development.

The large variation in the expressions of secondary metabolite biosynthetic genes is believed to have a major impact on the flavor of the varieties of tea products. Consistent with our results, an early study showed that caffeine biosynthesis was concentrated in young leaves and decreased with leaf growth [25]. Our data and data from others provided a good reference point from which to design, formulate, and manufacture tea products for industrial practice.

Caffeine accumulates in seeds and is released into the soil, where it inhibits the germination of other seeds [8]. Our data indicated that the overall levels of caffeine, flavonoid, and theanine biosynthetic genes are slightly elevated in seeds. It is likely that flavonoids and theanine accumulate in the seeds; however, this process remains to be further studied to understand the biological roles of these molecules in tea plant germination.

A previous study reported that theanine was distributed in young seedlings and that it was synthesized more rapidly in the roots than in the other tissues [26]. Our results demonstrated a similar phenomenon in mature tea plants, expanding the known theanine biosynthesis activity to old leaves and flowers. However, a high expression level was detected in buds and actively growing leaves, and lower levels were found in old leaves and roots. This pattern of distribution is different from that of the seedlings. Another study showed that in mature tea plants, the theanine content was the highest in the shoots (buds, first leaf, and second leaf), followed by the young stems and seeds, with the mature leaves having the lowest levels [49]. This is consistent with our results, in which mature leaves and roots have lower expression levels compared with other tissues.

### Transcription factor regulation network of flavonoid, caffeine, and theanine biosynthesis in *C. sinensis*

Transcriptional control is an important mechanism for regulating secondary metabolite production in plant cells [50]. Some transcription factors (TFs) are known to be involved in the regulation of secondary metabolism, such as R2R3-MYB, basic helix-loop-helix (bHLH) proteins, AP2/ERF family proteins, WRKY, NAC, DOF, bZIP, HD-ZIP, and TFIIIA zinc finger TFs [50]. In this study, TFs from TAIR [51] were used to search for candidate TFs in *C. sinensis*, and 5676 unigenes were predicted (Additional file 2).

**Fig. 11:**
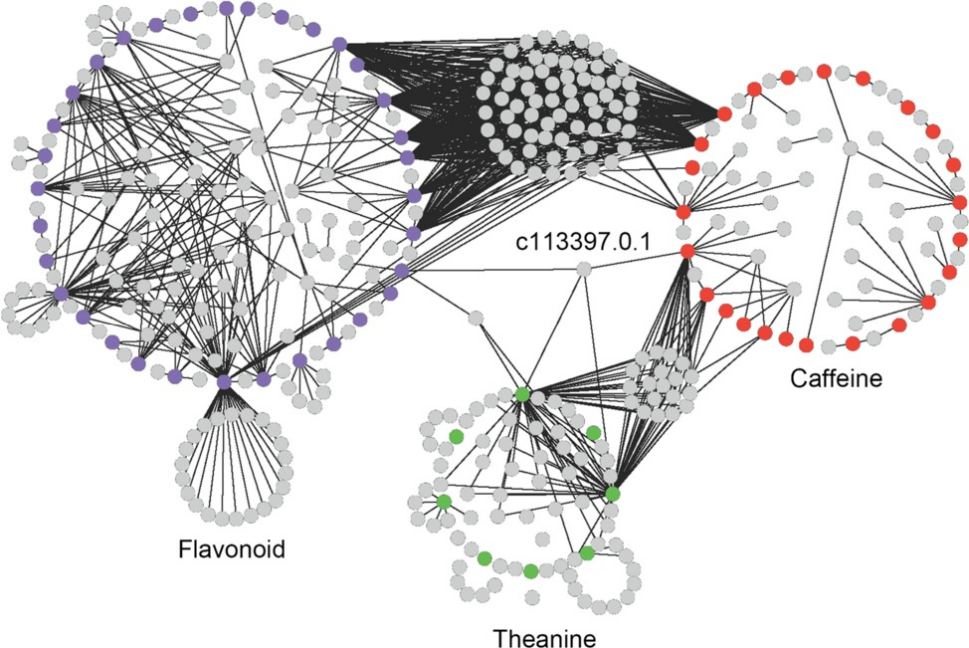
Transcription factor (TF) regulation network of the flavonoid, caffeine, and theanine biosynthesis pathways. The purple-, orange-, and green-colored nodes represent unigenes involved in the flavonoid, caffeine, and theanine biosynthesis pathways, respectively. Each gray node represents a TF, and a linked line between a pair of nodes denotes that their expressions are correlated

Using the expression profiles of the 13 examined tissues, a TF regulation network was created for the flavonoid, caffeine, and theanine biosynthetic pathways. The genes involved in these pathways and their associated TFs are illustrated in Fig. 11. A total of 339 TFs were identified in the network, belonging to 35 TF families. Importantly, many critical biosynthetic genes are associated with a number of TFs from different families, indicating that the transcriptional control of these biosynthetic pathways is complex.

In total, 206 TFs from 33 families were observed to be associated with 36 unigenes involved in flavonoid biosynthesis (Additional file 3). Among them, 95 and 76 TFs belonged to the MYB and bHLH families, respectively. Previous studies on flavonoid regulation in different plant species demonstrated the conservation of MYB/bHLH interactions in the control of flavonoid biosynthesis [52, 53]. MYB4 functions in the repression of *C4H* transcription in petunia [54]. Our work identified 26 TFs belonging to the MYB4 family, which were predicted to regulate the expression of *PAL* (c150940.1.1, c150940.1.3, c150940.1.10, and c150940.1.7), *F3H* (c155544.1.1), *F3′H* (c119661.0.1), and *FLS* (c166483.0.1). This result indicated that MYB4 might also regulate other important genes in the flavonoid biosynthetic pathway, in addition to *C4H*. In *Arabidopsis*, TT2 and TT8 were reported to regulate the flavonoid biosynthesis pathway [52, 53]. In our work, five unigenes (c129457.0.1, c159660.0.10, c159660.0.12, c159660.0.6, and c53663.0.1) encoding MYB TFs were homologous to TT2, and seven unigenes (c162621.0.12, c162621.0.13, c162621.0.4, c162621.0.7, c162621.0.9, c898672.0.1, and c135992.0.1) encoding bHLH TFs were homologous to TT8. However, the involvement of these genes in flavonoid biosynthesis was not confirmed in *C. sinensis*. These data point to possible differences in the mechanisms of regulation of flavonoid biosynthesis between *Arabidopsis* and *C. sinensis*.

In total, 132 TFs from 30 families correlated with the 24 unigenes in the caffeine biosynthesis pathway (Additional file 4). Most of these TFs belong to the bZIP, bHLH, and MYB families. AMPD is critical in the purine nucleotide pathway, and it plays an important role in the construction of the xanthene skeleton in caffeine biosynthesis. GATA and bHLH factors were reported to bind upstream of the *AMPD* transcription start site [55]. In the TF regulation network, there were 120 TFs related to the 12 *AMPD* unigenes. Among them, 27 and four TFs belonged to the bHLH and GATA families, respectively.

 There were 91 TFs associated with eight theanine biosynthetic genes (Additional file 5). These TFs were generally members of the AP2-EREBP, bHLH, C2H2, and WRKY families. *TS*/*GS* (c87293.0.1) was related to five TFs (c189005.0.1, c147239.0.14, c159134.1.3, c166979.0.1, c154617.0.2, c154617.0.1, and c124668.0.1). These TFs belonged to the Homeobox, MADS, C2C2-Gata, and C2H2 families. The other *TS*, c161936.0.8, might be regulated by ten TF unigenes (c163413.2.14, c163611.2.10, c163611.2.30, c57606.0.1, c152572.0.1, c159954.0.4, c147239.0.7, c135724.0.1, c156935.0.13, and c156935.0.3) that are members of the bZIP, C3H, MADS, REM, bHLH, and C2H2 families. In plants, TFs from the MADS family are closely related to seed, flower, and fruit formation [50]. We found that the MADS family of TFs might also play roles in the regulation of theanine biosynthesis.

Remarkably, the TF regulation network revealed the possible critical links in gene regulation between the flavonoid, caffeine, and theanine biosynthesis pathways (Fig. 11). A cluster of 67 TFs was associated with both the flavonoid and caffeine biosynthesis pathways. These TFs were linked to *PAL*, *C4H*, *F3H*, and *FLS* in the flavonoid pathway and to *APRT* and *AMPD* in the caffeine pathway. Conversely, a smaller group of 22 TFs was associated with both caffeine and theanine biosynthesis, linking to the two *AMPDs* in the caffeine biosynthetic pathway and to *ADC* and *GS* in the theanine pathway. Cross talk between the flavonoid and theanine biosynthetic pathways occurred through two TFs (c113397.0.1, c131731.0.2), which were linked to *C4H* in the flavonoid pathway and to *ADC* and *GS* in the theanine pathway. Notably, one TF (c113397.0.1) was linked to all three pathways via *C4H* (c136665.0.1) of the flavonoid pathway, *AMPD* (c163053.0.28) of the caffeine pathway, and the *ADC* (c156730.0.2) and *GS* unigenes (c163063.0.68, c163063.0.7, c163063.0.4) of the theanine pathway. This TF belonged to the NAC transcription factor family and was homologous to secondary wall-associated NAC domain protein 1 (*SND1*), which regulates secondary cell wall biosynthesis in *Arabidopsis* [56]. The above results not only pinpointed the possible critical check points in the flavonoid, caffeine, and theanine biosynthesis pathways, but also identified the candidate key transcription factors in the regulation of secondary metabolite biosynthesis and the possible mechanisms involved. For the first time, this work addresses the potentially important cross talk between the flavonoid, caffeine, and theanine biosynthesis pathways in *C. sinensis*.

## Conclusions

The tea plant (*C. sinensis*) is one of the most economically important beverage crops. To systematically study the gene expression pattern and the regulation of its secondary metabolic pathways during development and growth, we sampled the buds and leaves at various developmental stages, as well as the stems, flowers, seeds, and roots. RNA-seq was performed on each tissue, and transcriptome profiles were generated. We identified large sets of tissue-specific expressed genes in each of the 13 different organs/tissues. The expression patterns of genes involved in flavonoid, caffeine, and theanine biosynthesis were characterized, revealing the dynamic nature of the regulation of secondary metabolism during plant development and growth. Notably, the TF regulation network generated in this study revealed the possible critical links in gene regulation between the flavonoid, caffeine, and theanine biosynthesis pathways. This work not only aids our understanding of how the gene expression underlying secondary metabolic pathways is regulated during plant development and growth, but also provides an excellent reference for the design, formulation, and industrial manufacturing of tea products.

## Methods

### Plant materials

The tea plant [*Camellia sinensis* (L.) O. Kuntze cv. ‘*Longjing 43*’] was grown in the China National Germplasm Hangzhou Tea Repository of the Tea Research Institute, Chinese Academy of Agricultural Sciences. Thirteen samples from different tissues of 4-year-old tea plants were used in this study. The tissues sampled were as follows: apical bud, lateral bud at early stage, lateral bud, first leaf, second leaf, mature leaf, old leaf, one and a bud, two and a bud, stem, flower, seed, and root. Buds, first leaves, second leaves, and stems were collected on April 6, 2012; mature leaves, old leaves, and roots were collected on June 27, 2012; flowers and seeds were collected on November 17, 2012. The harvested samples were frozen immediately in liquid nitrogen and stored at − 70 °C in a freezer.

### Library construction and sequencing

Total RNA was extracted using the RNeasy Plus Mini kit (Qiagen, Valencia, CA, USA) from the 13 different tissues of the tea plant. The RNA integrity was confirmed using RNA 6000 Nano LabChips with a Bioanalyzer 2100 (Agilent Technologies, Palo Alto, CA, USA). The libraries for sequencing were prepared using a kit from Illumina (San Diego, CA, USA) and followed the manufacturer’s recommendations. Briefly, mRNA was purified from the total RNA (20 μg) using oligo(dT) magnetic beads, followed by fragmentation of the mRNA into small pieces using divalent cations under an elevated temperature. The cleaved RNA fragments were used for first-strand cDNA synthesis using reverse-transcriptase and random primers, followed by second-strand cDNA synthesis using DNA polymerase I and RNase H. After the end repair process and ligation of the adapters, the products were enriched by PCR to create the final cDNA library.

The cDNA libraries were sequenced from both the 5′ and 3′ ends on the Illumina HiSeq™ 2000 platform, according to the manufacturer’s instructions. The fluorescent image processing, base calling, and quality value calculation were performed by the Illumina data processing pipeline 1.4.

### Assembly of sequencing reads and data analysis

The image data output from the sequencer was transformed by base calling into sequence data, and these data are also referred to as raw data or raw reads. Usually, the raw reads contain some adapter sequences, ambiguous nucleotides (N) or low-quality bases, which will negatively affect subsequent analyses. Therefore, the raw reads with a proportion of ambiguous nucleotides larger than 5 % (*N* ≥ 5 %) or low-quality bases (more than 20 % nucleotides with quality value ≤ 10) were removed to obtain clean reads. De novo assembly was performed using the Trinity program (release 20130225 [27]). Trinity first combined the reads with a certain length of overlap to form longer fragments, which were called contigs. The reads could then be mapped back to contigs to obtain longer sequences using paired-end reads as a guide. Finally, Trinity connected the contigs and obtained the sequences that could not be extended on either end. Such sequences were defined as unigenes.

### Unigene functional annotation

All unigenes were used to search against the TAIR [30], Swiss-Prot [31], TrEMBL [32], COG [34], and Nr [29] databases using BLASTX algorithms with a threshold of E-value ≤ 10^−5^. The Rpstblastn program was used to search against the CDD database [33], and the E-value threshold was set to 10^−3^. Transcription factors from the TAIR database [51] were used to annotate the unigenes of *C. sinensis* using the Blastn program, with an E-value threshold of 10^−5^. The pathway analysis was carried out using KAAS (KEGG Automatic Annotation Server) [57]. Unigenes that mapped to the KEGG database were retained for detailed pathway analysis. GO classifications of all unigenes were collected on the basis of the annotated information from the Nr database, and the unigenes were annotated with three major GO categories (Cellular Component, Molecular Function, and Biological Process). GO terms that were enriched significantly in each tissue were obtained by BiNGO (v2.44) [58]. We performed enrichment analysis on our data using a hypergeometric test, with P-values corrected by the false discovery rate (Benjamini and Hochberg correlation). GO terms with corrected P-values of < 0.05 were considered to be significantly enriched.

### Expression profiling of all unigenes and unigenes involved in secondary metabolite biosynthesis

Reads were mapped against the assembled reference transcriptome for each sample using Bowtie2 (version2.1.0) [28]. The reads were permitted to map to multiple locations, but the best mapping site was used randomly in the downstream analysis. RPKM (reads per kilobase per million reads) was used to quantify gene expression, which can eliminate the effect of sequence length [59]. The RPKM value was calculated for every unigene in each tissue, and the log RPKM values for the unigenes involved in the flavonoid, caffeine, and theanine biosynthetic pathways in tea plants were obtained. The unigenes were clustered hierarchically in each secondary metabolite pathway according to their log RPKM values.

The expression level (in RPKM) of each unigene in each secondary metabolite biosynthesis pathway was ranked among the 13 tissues. An average ranking of all unigenes for a tissue was obtained by dividing the sum of all unigene rankings by the number of unigenes in the pathway. For each tissue, the average ranking of all unigenes from a biosynthetic pathway represented the relative expression strength of that pathway.

### Quantitative real-time PCR analysis

Total RNA was isolated from apical bud, lateral bud at early stage, lateral bud, one and a bud, two and a bud, first leaf, second leaf, mature leaf, old leaf, root, stem, flower, and seed tissues using an RNeasy Plus Mini kit (Qiagen). The RNA samples were treated with TURBO DNase (Ambion, Austin, TX, USA) at a concentration of 1.5 units/μg of total RNA prior to cDNA synthesis. An aliquot of 1 μg of total RNA was converted into first-strand cDNA via a reverse-transcription reaction with random hexamer primers and MultiScribe Reverse Transcriptase from a High Capacity cDNA Reverse Transcription Kit (Applied Biosystems, Foster City, CA, USA). The cDNA products were then diluted 10-fold with nuclease-free deionized water before being used as a template for real-time PCR. cDNA was amplified using SsoFast EvaGreen Supermix (Bio-Rad, Hercules, CA, USA) in a volume of 20 μL. The reaction mixture contained 10 μL of SsoFast EvaGreen Supermix, 5 μM each of the forward and reverse primers, and 2 μL of template cDNA. The PCR amplification was performed at an annealing temperature of 60 °C with an ABI 7500 real-time PCR system (Applied Biosystems) according to the manufacturer’s instructions. All of the analyzed unigenes were tested with three biological replicates and three technical replicates. The relative transcript abundances were calculated by the comparative cycle threshold method with the 18S ribosomal RNA gene as an internal standard. The primer pairs used for RT-PCR are listed in Additional file 6.

### Generation of the TF regulation network of the flavonoid, caffeine, and theanine biosynthesis pathways

All TF unigenes that were annotated in the TAIR database and the unigenes from the flavonoid, caffeine, and theanine biosynthesis pathways were selected for the generation of the TF regulation network. The Pearson coefficient was computed between any two unigenes based on their RPKM values from the 13 different tissues using R (version 2.15, function: cor). Unigene pairs with a coefficient ≥ 0.9 were considered to have correlated gene expression and were retained to build the TF regulation network. The correlation between two genes was denoted by a linked line in the network. The network was displayed using Cytoscape [50]. All TFs that were directly connected to a flavonoid, caffeine, or theanine biosynthetic gene were considered potential TFs underlying the regulation of biosynthesis.

### Availability of supporting data

The RNA-seq data from the 13 samples have been deposited in the NCBI Sequencing Read Archive database and can be accessed with the following accession numbers: SRR1053623, SRR1051214, SRR1054007, SRR1055110, SRR1055182, SRR1054086, SRR1054152, SRR1055108, SRR1055109, SRR1055932, SRR1055933, SRR1055934, and SRR1055944.

## Additional files

Additional file 1:
**GO terms of all of the unigenes and tissue-specific unigenes in each sample. **A DOCX document containing the GO enrichment classifications of the unigenes in each tissue and the tissue-specific unigenes. (DOC 1357 kb) Additional file 2:
**Transcription factor unigenes predicted in** *C. sinensis.* An XLSX document containing a list of transcription factor (TF) unigenes in *C. sinensis*. (XLSX 198 kb) Additional file 3:
**Transcription factor unigenes involved in flavonoid biosynthesis.** An XLSX document containing a list of transcription factor (TF) unigenes involved in flavonoid biosynthesis. (XLSX 23 kb) Additional file 4:
**Transcription factor unigenes involved in caffeine biosynthesis.** An XLSX document containing a list of transcription factor (TF) unigenes involved in caffeine biosynthesis. (XLSX 15 kb) Additional file 5:
**Transcription factor unigenes involved in theanine biosynthesis.** An XLSX document containing a list of transcription factor (TF) unigenes involved in theanine biosynthesis. (XLSX 17 kb) Additional file 6:
**List of primers used for RT-PCR verification.** A DOCX document containing a list of primers used for RT-PCR verification. (XLSX 16 kb)

## Abbreviations

*C. sinensis*: *Camellia sinensis*
EST: Expressed sequence tag
RNA-seq: RNA sequencing
1st leaf: First leaf
2nd leaf: Second leaf
one and a bud: One leaf and one bud
two and a bud: Two leaves and one bud
bp: Base pair
Mbp: Megabase pairs
Gbp: Gigabase pairs
NCBI: National Center for Biotechnology Information
Nr: NCBI non-redundant protein
TAIR: The Arabidopsis Information Resource
TrEMBL: Translated EMBL nucleotide sequence database
CDD: Conserved domain database
GO: Gene ontology
COG: Cluster of orthologous groups
PA: Proanthocyanidins
KEGG: Kyoto encyclopedia of genes and genomes
PAL: Phenylalanine ammonia-lyase
C4H: Cinnamic acid 4-hydroxylase
4CL: 4-coumarate-CoA ligase
CHS: Chalcone synthase
CHI: Chalcone isomerase
F3H: Flavanone 3-hydroxylase
F3′,5′H: Flavonoid 3′,5′-hydroxylase
F3′H: Flavonoid 3′-hydroxylase
FLS: Flavonol synthase
DFR: Dihydroflavonol 4-reductase
ANS: Anthocyanidin synthase
ANR: Anthocyanidin reductase
LAR: Leucocyanidin reductase
FGS: Flavan-3-ol gallate synthase
LDOX: Leucoanthocyanidin oxidase
UFGT: UDP-glucose: flavonoid 3-*O*-glucosyl transferase
Anase: Adenosine nucleosidase
APRT: Adenine phosphoribosyltransferase
AMPD: AMP deaminase
IMPDH: IMP dehydrogenase
5′-Nase: 5′-nucleotidase
7-NMT: 7-methylxanthosine synthase
N-MeNase: N-methylnucleotidase
MXMT: Theobromine synthase
TCS: Tea caffeine synthase
GS: Glutamine synthetase
GLS: Glutaminase
ALT: Alanine aminotransferase
ADC: Arginine decarboxylase
TS: Theanine synthetase
qRT-PCR: Quantitative real-time PCR
TF: Transcription factor
AtTFDB: Arabidopsis transcription factor database
