# Supplementary material for: Global transcriptome and gene regulation network for secondary metabolite biosynthesis of tea plant (Camellia sinensis)
Source: BMC Genomics. 2015 Jul 29;16(1):560. doi: 10.1186/s12864-015-1773-0 (PMC4518527; doi:10.1186/s12864-015-1773-0)
Supplement: Additional file 1: — GO terms of all of the unigenes and tissue-specific unigenes in each sample. A DOCX document containing the GO enrichment classifications of the unigenes in each tissue and the tissue-specific unigenes. (DOC 1357 kb) [file 12864_2015_1773_MOESM1_ESM.doc]

**Table S1. GO enrichment results of all unigenes in thirteen tissues from *C. sinensis*.**

| **GO-ID** | **p-value** | **corr p-value** | **Description** |
| --- | --- | --- | --- |
| **Apical Bud** | | | |
| GO:0044464 | 2.13E-08 | 5.57E-05 | cell part |
| GO:0005623 | 2.13E-08 | 5.57E-05 | cell |
| GO:0043412 | 1.68E-07 | 2.50E-04 | macromolecule modification |
| GO:0005575 | 1.92E-07 | 2.50E-04 | cellular_component |
| GO:0006464 | 1.28E-06 | 1.34E-03 | protein modification process |
| GO:0032440 | 2.27E-06 | 1.96E-03 | 2-alkenal reductase activity |
| GO:0043687 | 2.63E-06 | 1.96E-03 | post-translational protein modification |
| GO:0016773 | 4.32E-06 | 2.82E-03 | phosphotransferase activity, alcohol group as acceptor |
| GO:0005524 | 6.35E-06 | 3.68E-03 | ATP binding |
| GO:0031072 | 1.64E-05 | 7.84E-03 | heat shock protein binding |
| GO:0051276 | 1.65E-05 | 7.84E-03 | chromosome organization |
| GO:0006325 | 1.86E-05 | 8.10E-03 | chromatin organization |
| GO:0006996 | 2.13E-05 | 8.46E-03 | organelle organization |
| GO:0016020 | 2.27E-05 | 8.46E-03 | membrane |
| GO:0016628 | 3.71E-05 | 1.29E-02 | oxidoreductase activity, acting on the CH-CH group of donors, NAD or NADP as acceptor |
| GO:0046873 | 4.39E-05 | 1.43E-02 | metal ion transmembrane transporter activity |
| GO:0015079 | 5.06E-05 | 1.50E-02 | potassium ion transmembrane transporter activity |
| GO:0016301 | 5.30E-05 | 1.50E-02 | kinase activity |
| GO:0044427 | 5.47E-05 | 1.50E-02 | chromosomal part |
| GO:0004674 | 6.14E-05 | 1.60E-02 | protein serine/threonine kinase activity |
| GO:0042578 | 7.28E-05 | 1.81E-02 | phosphoric ester hydrolase activity |
| GO:0006468 | 8.32E-05 | 1.97E-02 | protein amino acid phosphorylation |
| GO:0004672 | 1.02E-04 | 2.31E-02 | protein kinase activity |
| GO:0005694 | 1.16E-04 | 2.53E-02 | chromosome |
| GO:0000785 | 2.16E-04 | 4.51E-02 | chromatin |
| **Lateral bud at early stage** | | |  |
| GO:0006259 | 2.74E-58 | 1.36E-54 | DNA metabolic process |
| GO:0003964 | 1.36E-36 | 3.39E-33 | RNA-directed DNA polymerase activity |
| GO:0034061 | 2.41E-36 | 4.00E-33 | DNA polymerase activity |
| GO:0006278 | 1.07E-34 | 1.33E-31 | RNA-dependent DNA replication |
| GO:0015074 | 4.61E-33 | 4.59E-30 | DNA integration |
| GO:0003676 | 3.16E-32 | 2.62E-29 | nucleic acid binding |
| GO:0006260 | 7.41E-32 | 5.27E-29 | DNA replication |
| GO:0016779 | 8.81E-30 | 5.48E-27 | nucleotidyltransferase activity |
| GO:0090304 | 3.15E-29 | 1.74E-26 | nucleic acid metabolic process |
| GO:0003723 | 3.21E-27 | 1.60E-24 | RNA binding |
| GO:0008270 | 6.94E-24 | 3.14E-21 | zinc ion ®¡ãing |
| GO:0016772 | 2.45E-19 | 1.02E-16 | transferase activity, transferring phosphorus-containing groups |
| GO:0006139 | 4.81E-18 | 1.84E-15 | nucleobase, nucleoside, nucleotide and nucleic acid metabolic process |
| GO:0016788 | 7.07E-14 | 2.51E-11 | hydrolase activity, acting on ester bonds |
| GO:0004518 | 1.83E-13 | 6.07E-11 | nuclease activity |
| GO:0004519 | 2.08E-11 | 6.48E-09 | endonuclease activity |
| GO:0034641 | 3.15E-09 | 9.22E-07 | cellular nitrogen compound metabolic process |
| GO:0006807 | 4.37E-08 | 1.21E-05 | nitrogen compound metabolic process |
| GO:0016740 | 8.15E-08 | 2.13E-05 | transferase activity |
| GO:0016893 | 1.44E-07 | 3.59E-05 | endonuclease activity, active with either ribo- or deoxyribonucleic acids and producing 5'-phosphomonoesters |
| GO:0044260 | 7.09E-07 | 1.68E-04 | cellular macromolecule metabolic process |
| GO:0004540 | 7.62E-07 | 1.72E-04 | ribonuclease activity |
| GO:0016891 | 1.21E-06 | 2.61E-04 | endoribonuclease activity, producing 5'-phosphomonoesters |
| GO:0043170 | 5.80E-06 | 1.19E-03 | macromolecule metabolic process |
| GO:0004521 | 5.97E-06 | 1.19E-03 | endoribonuclease activity |
| GO:0005488 | 7.85E-06 | 1.50E-03 | binding |
| GO:0004523 | 1.02E-05 | 1.88E-03 | ribonuclease H activity |
| GO:0090305 | 3.14E-05 | 5.57E-03 | nucleic acid phosphodiester bond hydrolysis |
| GO:0031072 | 5.71E-05 | 9.79E-03 | heat shock protein binding |
| GO:0032440 | 7.01E-05 | 1.16E-02 | 2-alkenal reductase activity |
| **Lateral bud** |  |  |  |
| GO:0008270 | 7.15E-08 | 3.57E-04 | zinc ion binding |
| GO:0031072 | 7.77E-06 | 1.46E-02 | heat shock protein binding |
| GO:0032440 | 8.77E-06 | 1.46E-02 | 2-alkenal reductase activity |
| GO:0043412 | 1.78E-05 | 2.22E-02 | macromolecule modification |
| **One and a bud** |  |  |  |
| GO:0043412 | 1.05E-09 | 5.30E-06 | macromolecule modification |
| GO:0016773 | 2.41E-09 | 6.06E-06 | phosphotransferase activity, alcohol group as acceptor |
| GO:0043687 | 1.53E-08 | 2.27E-05 | post-translational protein modification |
| GO:0006464 | 1.80E-08 | 2.27E-05 | protein modification process |
| GO:0006468 | 1.03E-07 | 1.04E-04 | protein amino acid phosphorylation |
| GO:0016301 | 1.32E-07 | 1.10E-04 | kinase activity |
| GO:0004672 | 1.52E-07 | 1.10E-04 | protein kinase activity |
| GO:0004674 | 5.77E-07 | 3.52E-04 | protein serine/threonine kinase activity |
| GO:0008270 | 6.29E-07 | 3.52E-04 | zinc ion binding |
| GO:0005524 | 1.63E-05 | 8.18E-03 | ATP binding |
| GO:0032440 | 2.24E-05 | 1.02E-02 | 2-alkenal reductase activity |
| GO:0006796 | 4.79E-05 | 1.86E-02 | phosphate metabolic process |
| GO:0006793 | 4.81E-05 | 1.86E-02 | phosphorus metabolic process |
| GO:0004697 | 5.30E-05 | 1.90E-02 | protein kinase C activity |
| GO:0031072 | 5.80E-05 | 1.94E-02 | heat shock protein binding |
| GO:0046873 | 9.93E-05 | 3.12E-02 | metal ion transmembrane transporter activity |
| **Two and a bud** |  |  |  |
| GO:0031072 | 3.08E-06 | 1.53E-02 | heat shock protein binding |
| GO:0032440 | 7.09E-06 | 1.76E-02 | 2-alkenal reductase activity |
| GO:0043412 | 1.33E-05 | 2.21E-02 | macromolecule modification |
| GO:0046873 | 2.94E-05 | 3.65E-02 | metal ion transmembrane transporter activity |
| **1st leaf** |  |  |  |
| GO:0043412 | 5.42E-11 | 2.68E-07 | macromolecule modification |
| GO:0006464 | 5.12E-10 | 1.27E-06 | protein modification process |
| GO:0016773 | 1.18E-09 | 1.95E-06 | phosphotransferase activity, alcohol group as acceptor |
| GO:0043687 | 1.64E-09 | 2.04E-06 | post-translational protein modification |
| GO:0016301 | 1.12E-08 | 1.11E-05 | kinase activity |
| GO:0006468 | 6.37E-08 | 4.58E-05 | protein amino acid phosphorylation |
| GO:0031072 | 7.04E-08 | 4.58E-05 | heat shock protein binding |
| GO:0004672 | 7.39E-08 | 4.58E-05 | protein kinase activity |
| GO:0004674 | 1.86E-07 | 1.02E-04 | protein serine/threonine kinase activity |
| GO:0032440 | 1.14E-06 | 5.66E-04 | 2-alkenal reductase activity |
| GO:0005524 | 1.83E-06 | 8.23E-04 | ATP binding |
| GO:0006793 | 4.54E-06 | 1.75E-03 | phosphorus metabolic process |
| GO:0006796 | 4.60E-06 | 1.75E-03 | phosphate metabolic process |
| GO:0046873 | 7.87E-06 | 2.78E-03 | metal ion transmembrane transporter activity |
| GO:0016020 | 4.68E-05 | 1.54E-02 | membrane |
| GO:0016310 | 5.65E-05 | 1.68E-02 | phosphorylation |
| GO:0015079 | 5.78E-05 | 1.68E-02 | potassium ion transmembrane transporter activity |
| GO:0042578 | 6.74E-05 | 1.80E-02 | phosphoric ester hydrolase activity |
| GO:0000186 | 6.91E-05 | 1.80E-02 | activation of MAPKK activity |
| GO:0016628 | 1.09E-04 | 2.69E-02 | oxidoreductase activity, acting on the CH-CH group of donors, NAD or NADP as acceptor |
| GO:0004709 | 1.15E-04 | 2.70E-02 | MAP kinase kinase kinase activity |
| GO:0044464 | 1.79E-04 | 3.86E-02 | cell part |
| GO:0005623 | 1.79E-04 | 3.86E-02 | cell |
| GO:0032147 | 2.34E-04 | 4.83E-02 | activation of protein kinase activity |
| **2nd leaf** |  |  |  |
| GO:0008104 | 2.83E-11 | 1.13E-07 | protein localization |
| GO:0031072 | 5.72E-11 | 1.13E-07 | heat shock protein binding |
| GO:0015031 | 8.36E-11 | 1.13E-07 | protein transport |
| GO:0045184 | 8.36E-11 | 1.13E-07 | establishment of protein localization |
| GO:0005524 | 3.83E-10 | 4.12E-07 | ATP binding |
| GO:0016791 | 1.48E-09 | 1.33E-06 | phosphatase activity |
| GO:0042578 | 1.09E-08 | 8.40E-06 | phosphoric ester hydrolase activity |
| GO:0004721 | 1.34E-08 | 9.05E-06 | phosphoprotein phosphatase activity |
| GO:0043412 | 2.86E-08 | 1.71E-05 | macromolecule modification |
| GO:0006886 | 8.39E-08 | 4.05E-05 | intracellular protein transport |
| GO:0034613 | 9.22E-08 | 4.05E-05 | cellular protein localization |
| GO:0046907 | 1.01E-07 | 4.05E-05 | intracellular transport |
| GO:0030163 | 1.24E-07 | 4.05E-05 | protein catabolic process |
| GO:0044265 | 1.27E-07 | 4.05E-05 | cellular macromolecule catabolic process |
| GO:0043632 | 1.28E-07 | 4.05E-05 | modification-dependent macromolecule catabolic process |
| GO:0019941 | 1.28E-07 | 4.05E-05 | modification-dependent protein catabolic process |
| GO:0006511 | 1.28E-07 | 4.05E-05 | ubiquitin-dependent protein catabolic process |
| GO:0006464 | 1.46E-07 | 4.35E-05 | protein modification process |
| GO:0033036 | 1.60E-07 | 4.35E-05 | macromolecule localization |
| GO:0044257 | 1.62E-07 | 4.35E-05 | cellular protein catabolic process |
| GO:0070727 | 3.13E-07 | 8.02E-05 | cellular macromolecule localization |
| GO:0044464 | 3.81E-07 | 8.93E-05 | cell part |
| GO:0005623 | 3.81E-07 | 8.93E-05 | cell |
| GO:0006996 | 3.99E-07 | 8.96E-05 | organelle organization |
| GO:0000166 | 4.47E-07 | 9.63E-05 | nucleotide binding |
| GO:0051603 | 4.68E-07 | 9.69E-05 | proteolysis involved in cellular protein catabolic process |
| GO:0003777 | 5.52E-07 | 1.07E-04 | microtubule motor activity |
| GO:0004221 | 5.58E-07 | 1.07E-04 | ubiquitin thiolesterase activity |
| GO:0023052 | 6.37E-07 | 1.18E-04 | signaling |
| GO:0051649 | 7.32E-07 | 1.31E-04 | establishment of localization in cell |
| GO:0016817 | 7.68E-07 | 1.34E-04 | hydrolase activity, acting on acid anhydrides |
| GO:0016568 | 9.12E-07 | 1.53E-04 | chromatin modification |
| GO:0051641 | 1.01E-06 | 1.65E-04 | cellular localization |
| GO:0016874 | 1.20E-06 | 1.90E-04 | ligase activity |
| GO:0016570 | 1.56E-06 | 2.40E-04 | histone modification |
| GO:0016311 | 1.63E-06 | 2.43E-04 | dephosphorylation |
| GO:0016818 | 2.09E-06 | 3.04E-04 | hydrolase activity, acting on acid anhydrides, in phosphorus-containing anhydrides |
| GO:0007165 | 2.53E-06 | 3.59E-04 | signal transduction |
| GO:0043687 | 2.81E-06 | 3.88E-04 | post-translational protein modification |
| GO:0016192 | 3.02E-06 | 4.07E-04 | vesicle-mediated transport |
| GO:0017111 | 3.18E-06 | 4.12E-04 | nucleoside-triphosphatase activity |
| GO:0006325 | 3.22E-06 | 4.12E-04 | chromatin organization |
| GO:0023060 | 3.56E-06 | 4.35E-04 | signal transmission |
| GO:0023046 | 3.56E-06 | 4.35E-04 | signaling process |
| GO:0051276 | 4.32E-06 | 5.18E-04 | chromosome organization |
| GO:0016569 | 5.44E-06 | 6.09E-04 | covalent chromatin modification |
| GO:0043039 | 5.47E-06 | 6.09E-04 | tRNA aminoacylation |
| GO:0043038 | 5.47E-06 | 6.09E-04 | amino acid activation |
| GO:0016462 | 5.58E-06 | 6.09E-04 | pyrophosphatase activity |
| GO:0034660 | 5.83E-06 | 6.09E-04 | ncRNA metabolic process |
| GO:0016875 | 6.04E-06 | 6.09E-04 | ligase activity, forming carbon-oxygen bonds |
| GO:0016876 | 6.04E-06 | 6.09E-04 | ligase activity, forming aminoacyl-tRNA and related compounds |
| GO:0004812 | 6.04E-06 | 6.09E-04 | aminoacyl-tRNA ligase activity |
| GO:0006470 | 6.18E-06 | 6.09E-04 | protein amino acid dephosphorylation |
| GO:0005634 | 6.22E-06 | 6.09E-04 | nucleus |
| GO:0006396 | 8.20E-06 | 7.87E-04 | RNA processing |
| GO:0023033 | 8.33E-06 | 7.87E-04 | signaling pathway |
| GO:0016773 | 9.16E-06 | 8.38E-04 | phosphotransferase activity, alcohol group as acceptor |
| GO:0043085 | 9.34E-06 | 8.38E-04 | positive regulation of catalytic activity |
| GO:0006418 | 9.34E-06 | 8.38E-04 | tRNA aminoacylation for protein translation |
| GO:0042623 | 1.04E-05 | 9.21E-04 | ATPase activity, coupled |
| GO:0004715 | 1.13E-05 | 9.83E-04 | non-membrane spanning protein tyrosine kinase activity |
| GO:0003774 | 1.88E-05 | 1.61E-03 | motor activity |
| GO:0004386 | 2.20E-05 | 1.85E-03 | helicase activity |
| GO:0005575 | 2.31E-05 | 1.91E-03 | cellular_component |
| GO:0016627 | 2.42E-05 | 1.98E-03 | oxidoreductase activity, acting on the CH-CH group of donors |
| GO:0006399 | 2.46E-05 | 1.98E-03 | tRNA metabolic process |
| GO:0050789 | 2.75E-05 | 2.18E-03 | regulation of biological process |
| GO:0032440 | 2.94E-05 | 2.29E-03 | 2-alkenal reductase activity |
| GO:0044093 | 3.04E-05 | 2.34E-03 | positive regulation of molecular function |
| GO:0070035 | 5.49E-05 | 4.06E-03 | purine NTP-dependent helicase activity |
| GO:0008026 | 5.49E-05 | 4.06E-03 | ATP-dependent helicase activity |
| GO:0016301 | 5.50E-05 | 4.06E-03 | kinase activity |
| GO:0023034 | 5.72E-05 | 4.16E-03 | intracellular signaling pathway |
| GO:0008287 | 5.81E-05 | 4.17E-03 | protein serine/threonine phosphatase complex |
| GO:0043234 | 6.19E-05 | 4.39E-03 | protein complex |
| GO:0001883 | 6.45E-05 | 4.51E-03 | purine nucleoside binding |
| GO:0032147 | 6.64E-05 | 4.58E-03 | activation of protein kinase activity |
| GO:0060589 | 6.81E-05 | 4.64E-03 | nucleoside-triphosphatase regulator activity |
| GO:0006352 | 6.92E-05 | 4.66E-03 | transcription initiation |
| GO:0030554 | 7.17E-05 | 4.77E-03 | adenyl nucleotide binding |
| GO:0032559 | 7.99E-05 | 5.25E-03 | adenyl ribonucleotide binding |
| GO:0009057 | 9.03E-05 | 5.86E-03 | macromolecule catabolic process |
| GO:0035556 | 1.11E-04 | 7.10E-03 | intracellular signal transduction |
| GO:0033674 | 1.20E-04 | 7.45E-03 | positive regulation of kinase activity |
| GO:0045860 | 1.20E-04 | 7.45E-03 | positive regulation of protein kinase activity |
| GO:0017076 | 1.20E-04 | 7.45E-03 | purine nucleotide binding |
| GO:0005654 | 1.27E-04 | 7.77E-03 | nucleoplasm |
| GO:0048580 | 1.29E-04 | 7.82E-03 | regulation of post-embryonic development |
| GO:0032555 | 1.39E-04 | 8.21E-03 | purine ribonucleotide binding |
| GO:0032553 | 1.39E-04 | 8.21E-03 | ribonucleotide binding |
| GO:0051347 | 1.42E-04 | 8.32E-03 | positive regulation of transferase activity |
| GO:0004722 | 1.51E-04 | 8.75E-03 | protein serine/threonine phosphatase activity |
| GO:0050794 | 1.73E-04 | 9.92E-03 | regulation of cellular process |
| GO:0016887 | 1.75E-04 | 9.92E-03 | ATPase activity |
| GO:0051188 | 1.86E-04 | 1.04E-02 | cofactor biosynthetic process |
| GO:0016573 | 2.09E-04 | 1.16E-02 | histone acetylation |
| GO:0016790 | 2.27E-04 | 1.25E-02 | thiolester hydrolase activity |
| GO:0043543 | 2.33E-04 | 1.27E-02 | protein amino acid acylation |
| GO:0012505 | 2.50E-04 | 1.35E-02 | endomembrane system |
| GO:0030695 | 2.53E-04 | 1.35E-02 | GTPase regulator activity |
| GO:0006457 | 2.80E-04 | 1.47E-02 | protein folding |
| GO:0005794 | 2.81E-04 | 1.47E-02 | Golgi apparatus |
| GO:0030117 | 3.30E-04 | 1.69E-02 | membrane coat |
| GO:0048475 | 3.30E-04 | 1.69E-02 | coated membrane |
| GO:0006888 | 3.42E-04 | 1.74E-02 | ER to Golgi vesicle-mediated transport |
| GO:0007186 | 3.81E-04 | 1.92E-02 | G-protein coupled receptor protein signaling pathway |
| GO:0009894 | 4.00E-04 | 2.00E-02 | regulation of catabolic process |
| GO:0016811 | 4.05E-04 | 2.00E-02 | hydrolase activity, acting on carbon-nitrogen (but not peptide) bonds, in linear amides |
| GO:0009787 | 4.18E-04 | 2.05E-02 | regulation of abscisic acid mediated signaling pathway |
| GO:0008238 | 4.29E-04 | 2.08E-02 | exopeptidase activity |
| GO:0051082 | 4.83E-04 | 2.32E-02 | unfolded protein binding |
| GO:0006473 | 5.18E-04 | 2.47E-02 | protein amino acid acetylation |
| GO:0044451 | 5.28E-04 | 2.49E-02 | nucleoplasm part |
| GO:0000186 | 5.31E-04 | 2.49E-02 | activation of MAPKK activity |
| GO:0046873 | 5.84E-04 | 2.71E-02 | metal ion transmembrane transporter activity |
| GO:0008237 | 6.06E-04 | 2.79E-02 | metallopeptidase activity |
| GO:0044428 | 6.21E-04 | 2.83E-02 | nuclear part |
| GO:0001882 | 6.30E-04 | 2.85E-02 | nucleoside binding |
| GO:0005388 | 7.92E-04 | 3.53E-02 | calcium-transporting ATPase activity |
| GO:0016628 | 7.93E-04 | 3.53E-02 | oxidoreductase activity, acting on the CH-CH group of donors, NAD or NADP as acceptor |
| GO:0008047 | 8.94E-04 | 3.95E-02 | enzyme activator activity |
| GO:0004672 | 9.75E-04 | 4.27E-02 | protein kinase activity |
| GO:0016810 | 1.00E-03 | 4.33E-02 | hydrolase activity, acting on carbon-nitrogen (but not peptide) bonds |
| GO:0015085 | 1.01E-03 | 4.33E-02 | calcium ion transmembrane transporter activity |
| GO:0004709 | 1.01E-03 | 4.33E-02 | MAP kinase kinase kinase activity |
| GO:0004402 | 1.13E-03 | 4.76E-02 | histone acetyltransferase activity |
| GO:0004468 | 1.13E-03 | 4.76E-02 | lysine N-acetyltransferase activity |
| **Mature leaf** |  |  |  |
| GO:0016773 | 1.75E-17 | 8.64E-14 | phosphotransferase activity, alcohol group as acceptor |
| GO:0043412 | 1.91E-16 | 4.35E-13 | macromolecule modification |
| GO:0043687 | 2.65E-16 | 4.35E-13 | post-translational protein modification |
| GO:0006464 | 4.96E-16 | 6.12E-13 | protein modification process |
| GO:0016301 | 1.44E-15 | 1.42E-12 | kinase activity |
| GO:0006468 | 1.80E-15 | 1.48E-12 | protein amino acid phosphorylation |
| GO:0004672 | 6.68E-15 | 4.71E-12 | protein kinase activity |
| GO:0004674 | 1.21E-13 | 7.45E-11 | protein serine/threonine kinase activity |
| GO:0006793 | 1.05E-12 | 5.28E-10 | phosphorus metabolic process |
| GO:0006796 | 1.07E-12 | 5.28E-10 | phosphate metabolic process |
| GO:0016310 | 1.80E-11 | 8.09E-09 | phosphorylation |
| GO:0031072 | 4.56E-08 | 1.87E-05 | heat shock protein binding |
| GO:0032440 | 1.54E-07 | 5.86E-05 | 2-alkenal reductase activity |
| GO:0005524 | 3.56E-07 | 1.25E-04 | ATP binding |
| GO:0016021 | 1.36E-06 | 4.48E-04 | integral to membrane |
| GO:0031224 | 2.06E-06 | 6.36E-04 | intrinsic to membrane |
| GO:0016020 | 2.50E-06 | 7.26E-04 | membrane |
| GO:0000166 | 7.57E-06 | 2.07E-03 | nucleotide binding |
| GO:0016628 | 2.56E-05 | 6.64E-03 | oxidoreductase activity, acting on the CH-CH group of donors, NAD or NADP as acceptor |
| GO:0046873 | 3.73E-05 | 9.20E-03 | metal ion transmembrane transporter activity |
| GO:0032559 | 7.32E-05 | 1.72E-02 | adenyl ribonucleotide binding |
| GO:0004713 | 9.15E-05 | 2.05E-02 | protein tyrosine kinase activity |
| GO:0044425 | 1.15E-04 | 2.43E-02 | membrane part |
| GO:0030554 | 1.22E-04 | 2.43E-02 | adenyl nucleotide binding |
| GO:0001883 | 1.30E-04 | 2.43E-02 | purine nucleoside binding |
| GO:0048544 | 1.33E-04 | 2.43E-02 | recognition of pollen |
| GO:0008037 | 1.33E-04 | 2.43E-02 | cell recognition |
| GO:0016627 | 1.50E-04 | 2.62E-02 | oxidoreductase activity, acting on the CH-CH group of donors |
| GO:0042578 | 1.54E-04 | 2.62E-02 | phosphoric ester hydrolase activity |
| GO:0032555 | 1.78E-04 | 2.83E-02 | purine ribonucleotide binding |
| GO:0032553 | 1.78E-04 | 2.83E-02 | ribonucleotide binding |
| GO:0019787 | 2.82E-04 | 4.29E-02 | small conjugating protein ligase activity |
| GO:0017076 | 2.87E-04 | 4.29E-02 | purine nucleotide binding |
| GO:0035556 | 2.97E-04 | 4.30E-02 | intracellular signal transduction |
| GO:0004842 | 3.31E-04 | 4.62E-02 | ubiquitin-protein ligase activity |
| GO:0060089 | 3.46E-04 | 4.62E-02 | molecular transducer activity |
| GO:0004871 | 3.46E-04 | 4.62E-02 | signal transducer activity |
| GO:0015932 | 3.80E-04 | 4.86E-02 | nucleobase, nucleoside, nucleotide and nucleic acid transmembrane transporter activity |
| GO:0009875 | 3.84E-04 | 4.86E-02 | pollen-pistil interaction |
| **Old leaf** |  |  |  |
| GO:0016773 | 1.35E-10 | 6.63E-07 | phosphotransferase activity, alcohol group as acceptor |
| GO:0043687 | 1.08E-09 | 1.88E-06 | post-translational protein modification |
| GO:0016772 | 1.39E-09 | 1.88E-06 | transferase activity, transferring phosphorus-containing groups |
| GO:0043412 | 1.53E-09 | 1.88E-06 | macromolecule modification |
| GO:0006464 | 2.69E-09 | 2.23E-06 | protein modification process |
| GO:0008270 | 3.06E-09 | 2.23E-06 | zinc ion binding |
| GO:0004674 | 3.18E-09 | 2.23E-06 | protein serine/threonine kinase activity |
| GO:0016301 | 4.65E-09 | 2.85E-06 | kinase activity |
| GO:0006468 | 1.13E-08 | 6.14E-06 | protein amino acid phosphorylation |
| GO:0004672 | 2.34E-08 | 1.15E-05 | protein kinase activity |
| GO:0004713 | 1.70E-07 | 7.59E-05 | protein tyrosine kinase activity |
| GO:0016788 | 2.50E-07 | 1.02E-04 | hydrolase activity, acting on ester bonds |
| GO:0048544 | 3.71E-07 | 1.30E-04 | recognition of pollen |
| GO:0008037 | 3.71E-07 | 1.30E-04 | cell recognition |
| GO:0006793 | 6.92E-07 | 2.14E-04 | phosphorus metabolic process |
| GO:0006796 | 6.98E-07 | 2.14E-04 | phosphate metabolic process |
| GO:0031072 | 1.12E-06 | 3.22E-04 | heat shock protein binding |
| GO:0009875 | 1.69E-06 | 4.61E-04 | pollen-pistil interaction |
| GO:0016310 | 3.61E-06 | 9.32E-04 | phosphorylation |
| GO:0004518 | 5.33E-06 | 1.31E-03 | nuclease activity |
| GO:0009960 | 7.72E-05 | 1.80E-02 | endosperm development |
| GO:0007154 | 8.46E-05 | 1.89E-02 | cell communication |
| GO:0006259 | 1.03E-04 | 2.20E-02 | DNA metabolic process |
| GO:0004519 | 1.11E-04 | 2.21E-02 | endonuclease activity |
| GO:0032440 | 1.13E-04 | 2.21E-02 | 2-alkenal reductase activity |
| GO:0016740 | 2.19E-04 | 4.12E-02 | transferase activity |
| GO:0042578 | 2.61E-04 | 4.73E-02 | phosphoric ester hydrolase activity |
| **Stem** |  |  |  |
| GO:0043412 | 5.71E-07 | 1.16E-03 | macromolecule modification |
| GO:0044464 | 6.48E-07 | 1.16E-03 | cell part |
| GO:0005623 | 6.48E-07 | 1.16E-03 | cell |
| GO:0016773 | 1.38E-06 | 1.85E-03 | phosphotransferase activity, alcohol group as acceptor |
| GO:0005575 | 3.00E-06 | 3.21E-03 | cellular_component |
| GO:0006464 | 4.05E-06 | 3.61E-03 | protein modification process |
| GO:0043687 | 5.79E-06 | 4.02E-03 | post-translational protein modification |
| GO:0005524 | 6.00E-06 | 4.02E-03 | ATP binding |
| GO:0016301 | 1.17E-05 | 6.97E-03 | kinase activity |
| GO:0042578 | 1.54E-05 | 8.24E-03 | phosphoric ester hydrolase activity |
| GO:0031072 | 2.21E-05 | 1.07E-02 | heat shock protein binding |
| GO:0006468 | 3.77E-05 | 1.68E-02 | protein amino acid phosphorylation |
| GO:0004672 | 8.36E-05 | 3.44E-02 | protein kinase activity |
| GO:0004674 | 1.24E-04 | 4.45E-02 | protein serine/threonine kinase activity |
| GO:0032440 | 1.25E-04 | 4.45E-02 | 2-alkenal reductase activity |
| **Flower** |  |  |  |
| GO:0044464 | 2.75E-09 | 7.21E-06 | cell part |
| GO:0005623 | 2.75E-09 | 7.21E-06 | cell |
| GO:0005575 | 9.12E-09 | 1.59E-05 | cellular_component |
| GO:0043412 | 1.48E-08 | 1.61E-05 | macromolecule modification |
| GO:0031072 | 1.72E-08 | 1.61E-05 | heat shock protein binding |
| GO:0006464 | 1.85E-08 | 1.61E-05 | protein modification process |
| GO:0043687 | 3.37E-08 | 2.52E-05 | post-translational protein modification |
| GO:0016773 | 2.10E-07 | 1.37E-04 | phosphotransferase activity, alcohol group as acceptor |
| GO:0016020 | 9.22E-07 | 5.37E-04 | membrane |
| GO:0044267 | 1.25E-06 | 6.57E-04 | cellular protein metabolic process |
| GO:0016301 | 2.72E-06 | 1.29E-03 | kinase activity |
| GO:0006468 | 4.28E-06 | 1.83E-03 | protein amino acid phosphorylation |
| GO:0004672 | 4.54E-06 | 1.83E-03 | protein kinase activity |
| GO:0019787 | 7.93E-06 | 2.97E-03 | small conjugating protein ligase activity |
| GO:0004842 | 9.49E-06 | 3.17E-03 | ubiquitin-protein ligase activity |
| GO:0004674 | 9.68E-06 | 3.17E-03 | protein serine/threonine kinase activity |
| GO:0015079 | 1.31E-05 | 4.05E-03 | potassium ion transmembrane transporter activity |
| GO:0046873 | 1.72E-05 | 5.00E-03 | metal ion transmembrane transporter activity |
| GO:0042578 | 3.02E-05 | 8.33E-03 | phosphoric ester hydrolase activity |
| GO:0019538 | 4.36E-05 | 1.14E-02 | protein metabolic process |
| GO:0032446 | 4.97E-05 | 1.24E-02 | protein modification by small protein conjugation |
| GO:0006793 | 8.15E-05 | 1.81E-02 | phosphorus metabolic process |
| GO:0006796 | 8.26E-05 | 1.81E-02 | phosphate metabolic process |
| GO:0016567 | 8.30E-05 | 1.81E-02 | protein ubiquitination |
| GO:0016791 | 1.18E-04 | 2.48E-02 | phosphatase activity |
| GO:0070647 | 1.28E-04 | 2.58E-02 | protein modification by small protein conjugation or removal |
| GO:0000151 | 1.42E-04 | 2.75E-02 | ubiquitin ligase complex |
| GO:0016881 | 2.46E-04 | 4.60E-02 | acid-amino acid ligase activity |
| GO:0015085 | 2.72E-04 | 4.91E-02 | calcium ion transmembrane transporter activity |
| **Seed** |  |  |  |
| GO:0016773 | 3.04E-07 | 1.50E-03 | phosphotransferase activity, alcohol group as acceptor |
| GO:0043412 | 1.58E-06 | 3.88E-03 | macromolecule modification |
| GO:0006468 | 3.66E-06 | 3.88E-03 | protein amino acid phosphorylation |
| GO:0032440 | 4.54E-06 | 3.88E-03 | 2-alkenal reductase activity |
| GO:0006464 | 4.69E-06 | 3.88E-03 | protein modification process |
| GO:0016301 | 4.70E-06 | 3.88E-03 | kinase activity |
| GO:0004672 | 6.06E-06 | 4.28E-03 | protein kinase activity |
| GO:0043687 | 7.27E-06 | 4.50E-03 | post-translational protein modification |
| GO:0004674 | 8.33E-06 | 4.58E-03 | protein serine/threonine kinase activity |
| GO:0031072 | 1.14E-05 | 5.64E-03 | heat shock protein binding |
| GO:0008270 | 1.99E-05 | 8.93E-03 | zinc ion binding |
| GO:0042578 | 6.29E-05 | 2.59E-02 | phosphoric ester hydrolase activity |
| GO:0015079 | 7.07E-05 | 2.69E-02 | potassium ion transmembrane transporter activity |
| GO:0016788 | 1.06E-04 | 3.73E-02 | hydrolase activity, acting on ester bonds |
| GO:0009960 | 1.24E-04 | 4.08E-02 | endosperm development |
| **Root** |  |  |  |
| GO:0006464 | 1.19E-08 | 3.40E-05 | protein modification process |
| GO:0043412 | 1.46E-08 | 3.40E-05 | macromolecule modification |
| GO:0043687 | 2.09E-08 | 3.40E-05 | post-translational protein modification |
| GO:0016773 | 5.40E-08 | 6.60E-05 | phosphotransferase activity, alcohol group as acceptor |
| GO:0006468 | 3.19E-07 | 2.86E-04 | protein amino acid phosphorylation |
| GO:0004672 | 3.50E-07 | 2.86E-04 | protein kinase activity |
| GO:0016301 | 4.94E-07 | 3.46E-04 | kinase activity |
| GO:0032440 | 7.53E-07 | 4.61E-04 | 2-alkenal reductase activity |
| GO:0004674 | 1.68E-06 | 9.13E-04 | protein serine/threonine kinase activity |
| GO:0031072 | 2.42E-06 | 1.15E-03 | heat shock protein binding |
| GO:0046873 | 2.58E-06 | 1.15E-03 | metal ion transmembrane transporter activity |
| GO:0016757 | 2.10E-05 | 8.57E-03 | transferase activity, transferring glycosyl groups |
| GO:0016706 | 3.72E-05 | 1.40E-02 | oxidoreductase activity, acting on paired donors, with incorporation or reduction of molecular oxygen, 2-oxoglutarate as one donor, and incorporation of one atom each of oxygen into both donors |
| GO:0019787 | 5.06E-05 | 1.63E-02 | small conjugating protein ligase activity |
| GO:0006796 | 5.24E-05 | 1.63E-02 | phosphate metabolic process |
| GO:0009960 | 5.37E-05 | 1.63E-02 | endosperm development |
| GO:0006793 | 5.99E-05 | 1.63E-02 | phosphorus metabolic process |
| GO:0004842 | 6.00E-05 | 1.63E-02 | ubiquitin-protein ligase activity |
| GO:0042578 | 6.74E-05 | 1.74E-02 | phosphoric ester hydrolase activity |
| GO:0016628 | 7.29E-05 | 1.79E-02 | oxidoreductase activity, acting on the CH-CH group of donors, NAD or NADP as acceptor |
| GO:0016021 | 9.19E-05 | 2.14E-02 | integral to membrane |
| GO:0004715 | 1.09E-04 | 2.43E-02 | non-membrane spanning protein tyrosine kinase activity |
| GO:0016407 | 1.18E-04 | 2.50E-02 | acetyltransferase activity |
| GO:0016791 | 1.29E-04 | 2.50E-02 | phosphatase activity |
| GO:0016627 | 1.34E-04 | 2.50E-02 | oxidoreductase activity, acting on the CH-CH group of donors |
| GO:0016020 | 1.36E-04 | 2.50E-02 | membrane |
| GO:0047134 | 1.40E-04 | 2.50E-02 | protein-disulfide reductase activity |
| GO:0031224 | 1.43E-04 | 2.50E-02 | intrinsic to membrane |
| GO:0016758 | 1.84E-04 | 3.00E-02 | transferase activity, transferring hexosyl groups |
| GO:0035556 | 1.85E-04 | 3.00E-02 | intracellular signal transduction |
| GO:0023034 | 1.90E-04 | 3.00E-02 | intracellular signaling pathway |
| GO:0032446 | 2.47E-04 | 3.78E-02 | protein modification by small protein conjugation |
| GO:0005388 | 3.38E-04 | 4.98E-02 | calcium-transporting ATPase activity |
| GO:0004697 | 3.46E-04 | 4.98E-02 | protein kinase C activity |

**Table S2. GO enrichment result of tissue specific unigenes from *C. sinensis*.**

| **GO-ID** | **p-value** | **corr p-value** | **Description** |
| --- | --- | --- | --- |
| **All buds** |  |  |  |
| GO:0006259 | 0.00E+00 | 0.00E+00 | DNA metabolic process |
| GO:0090304 | 1.78E-305 | 0.00E+00 | nucleic acid metabolic process |
| GO:0003964 | 6.05E-299 | 0.00E+00 | RNA-directed DNA polymerase activity |
| GO:0034061 | 3.65E-294 | 0.00E+00 | DNA polymerase activity |
| GO:0006278 | 1.43E-292 | 0.00E+00 | RNA-dependent DNA replication |
| GO:0006260 | 3.23E-272 | 0.00E+00 | DNA replication |
| GO:0003676 | 4.69E-263 | 0.00E+00 | nucleic acid binding |
| GO:0016779 | 5.36E-257 | 0.00E+00 | nucleotidyltransferase activity |
| GO:0006139 | 1.80E-248 | 0.00E+00 | nucleobase, nucleoside, nucleotide and nucleic acid metabolic process |
| GO:0015074 | 5.75E-230 | 0.00E+00 | DNA integration |
| GO:0034641 | 2.19E-210 | 0.00E+00 | cellular nitrogen compound metabolic process |
| GO:0003723 | 2.11E-207 | 0.00E+00 | RNA binding |
| GO:0006807 | 2.65E-203 | 0.00E+00 | nitrogen compound metabolic process |
| GO:0044260 | 2.53E-136 | 0.00E+00 | cellular macromolecule metabolic process |
| GO:0043170 | 9.45E-121 | 0.00E+00 | macromolecule metabolic process |
| GO:0016772 | 3.62E-110 | 0.00E+00 | transferase activity, transferring phosphorus-containing groups |
| GO:0034645 | 7.29E-100 | 7.88E-98 | cellular macromolecule biosynthetic process |
| GO:0009059 | 1.25E-99 | 1.28E-97 | macromolecule biosynthetic process |
| GO:0005488 | 1.47E-75 | 1.43E-73 | binding |
| GO:0044237 | 1.87E-71 | 1.72E-69 | cellular metabolic process |
| GO:0044238 | 9.53E-62 | 8.34E-60 | primary metabolic process |
| GO:0044249 | 9.91E-57 | 8.28E-55 | cellular biosynthetic process |
| GO:0016740 | 5.83E-53 | 4.66E-51 | transferase activity |
| GO:0008270 | 2.54E-51 | 1.94E-49 | zinc ion binding |
| GO:0009058 | 3.55E-50 | 2.61E-48 | biosynthetic process |
| GO:0004519 | 9.76E-46 | 6.90E-44 | endonuclease activity |
| GO:0009987 | 9.06E-45 | 6.17E-43 | cellular process |
| GO:0004518 | 1.98E-40 | 1.30E-38 | nuclease activity |
| GO:0004523 | 1.41E-39 | 8.92E-38 | ribonuclease H activity |
| GO:0016893 | 6.40E-38 | 3.92E-36 | endonuclease activity, active with either ribo- or deoxyribonucleic acids and producing 5'-phosphomonoesters |
| GO:0016891 | 1.45E-37 | 8.62E-36 | endoribonuclease activity, producing 5'-phosphomonoesters |
| GO:0004521 | 2.67E-36 | 1.54E-34 | endoribonuclease activity |
| GO:0004540 | 4.81E-34 | 2.68E-32 | ribonuclease activity |
| GO:0046914 | 1.11E-27 | 6.01E-26 | transition metal ion binding |
| GO:0008152 | 4.94E-27 | 2.59E-25 | metabolic process |
| GO:0016788 | 9.28E-15 | 4.74E-13 | hydrolase activity, acting on ester bonds |
| GO:0004190 | 5.40E-13 | 2.62E-11 | aspartic-type endopeptidase activity |
| GO:0070001 | 5.40E-13 | 2.62E-11 | aspartic-type peptidase activity |
| GO:0003674 | 6.26E-13 | 2.95E-11 | molecular_function |
| GO:0046872 | 6.48E-13 | 2.98E-11 | metal ion binding |
| GO:0043167 | 7.47E-11 | 3.27E-09 | ion binding |
| GO:0043169 | 7.47E-11 | 3.27E-09 | cation binding |
| GO:0004170 | 1.93E-07 | 7.54E-06 | dUTP diphosphatase activity |
| GO:0009211 | 1.93E-07 | 7.54E-06 | pyrimidine deoxyribonucleoside triphosphate metabolic process |
| GO:0009120 | 1.93E-07 | 7.54E-06 | deoxyribonucleoside metabolic process |
| GO:0046125 | 1.93E-07 | 7.54E-06 | pyrimidine deoxyribonucleoside metabolic process |
| GO:0046080 | 1.93E-07 | 7.54E-06 | dUTP metabolic process |
| GO:0047429 | 4.67E-07 | 1.79E-05 | nucleoside-triphosphate diphosphatase activity |
| GO:0015979 | 3.02E-06 | 1.13E-04 | photosynthesis |
| GO:0009200 | 5.87E-06 | 2.16E-04 | deoxyribonucleoside triphosphate metabolic process |
| GO:0006281 | 9.41E-06 | 3.29E-04 | DNA repair |
| GO:0009219 | 9.48E-06 | 3.29E-04 | pyrimidine deoxyribonucleotide metabolic process |
| GO:0009394 | 9.48E-06 | 3.29E-04 | 2'-deoxyribonucleotide metabolic process |
| GO:0006974 | 2.13E-05 | 7.24E-04 | response to DNA damage stimulus |
| GO:0009521 | 2.89E-04 | 9.65E-03 | photosystem |
| GO:0006298 | 3.03E-04 | 9.77E-03 | mismatch repair |
| GO:0030983 | 3.03E-04 | 9.77E-03 | mismatched DNA binding |
| GO:0016459 | 3.86E-04 | 1.22E-02 | myosin complex |
| GO:0009262 | 1.00E-03 | 3.12E-02 | deoxyribonucleotide metabolic process |
| GO:0004175 | 1.20E-03 | 3.68E-02 | endopeptidase activity |
| GO:0070161 | 1.37E-03 | 3.86E-02 | anchoring junction |
| GO:0030055 | 1.37E-03 | 3.86E-02 | cell-substrate junction |
| GO:0005912 | 1.37E-03 | 3.86E-02 | adherens junction |
| GO:0005925 | 1.37E-03 | 3.86E-02 | focal adhesion |
| GO:0005924 | 1.37E-03 | 3.86E-02 | cell-substrate adherens junction |
| GO:0033554 | 1.44E-03 | 4.02E-02 | cellular response to stress |
| GO:0015629 | 1.51E-03 | 4.15E-02 | actin cytoskeleton |
| **Bud and leaf** | |  |  |
| GO:0006259 | 4.53E-43 | 7.72E-40 | DNA metabolic process |
| GO:0034061 | 2.41E-40 | 2.06E-37 | DNA polymerase activity |
| GO:0006278 | 3.39E-39 | 1.92E-36 | RNA-dependent DNA replication |
| GO:0003964 | 7.82E-39 | 3.33E-36 | RNA-directed DNA polymerase activity |
| GO:0006260 | 6.60E-37 | 2.25E-34 | DNA replication |
| GO:0016779 | 5.34E-35 | 1.51E-32 | nucleotidyltransferase activity |
| GO:0003723 | 6.50E-34 | 1.58E-31 | RNA binding |
| GO:0090304 | 6.11E-30 | 1.30E-27 | nucleic acid metabolic process |
| GO:0044260 | 2.51E-23 | 4.74E-21 | cellular macromolecule metabolic process |
| GO:0006139 | 1.13E-22 | 1.93E-20 | nucleobase, nucleoside, nucleotide and nucleic acid metabolic process |
| GO:0009059 | 1.41E-22 | 2.18E-20 | macromolecule biosynthetic process |
| GO:0034645 | 2.54E-22 | 3.61E-20 | cellular macromolecule biosynthetic process |
| GO:0043170 | 8.15E-22 | 1.07E-19 | macromolecule metabolic process |
| GO:0016772 | 8.49E-19 | 1.03E-16 | transferase activity, transferring phosphorus-containing groups |
| GO:0034641 | 8.40E-17 | 9.54E-15 | cellular nitrogen compound metabolic process |
| GO:0003676 | 2.24E-16 | 2.38E-14 | nucleic acid binding |
| GO:0006807 | 2.44E-16 | 2.45E-14 | nitrogen compound metabolic process |
| GO:0044238 | 9.13E-11 | 8.63E-09 | primary metabolic process |
| GO:0044249 | 1.17E-10 | 1.04E-08 | cellular biosynthetic process |
| GO:0009058 | 1.22E-10 | 1.04E-08 | biosynthetic process |
| GO:0016740 | 2.60E-10 | 2.11E-08 | transferase activity |
| GO:0015074 | 1.54E-09 | 1.19E-07 | DNA integration |
| GO:0044237 | 4.59E-08 | 3.40E-06 | cellular metabolic process |
| GO:0005840 | 6.32E-08 | 4.48E-06 | ribosome |
| GO:0003735 | 7.12E-08 | 4.85E-06 | structural constituent of ribosome |
| GO:0030529 | 8.72E-07 | 5.71E-05 | ribonucleoprotein complex |
| GO:0004519 | 1.07E-06 | 6.77E-05 | endonuclease activity |
| GO:0004518 | 2.82E-06 | 1.71E-04 | nuclease activity |
| GO:0009987 | 4.79E-06 | 2.81E-04 | cellular process |
| GO:0006281 | 7.34E-06 | 4.17E-04 | DNA repair |
| GO:0006974 | 1.43E-05 | 7.81E-04 | response to DNA damage stimulus |
| GO:0000922 | 1.47E-05 | 7.81E-04 | spindle pole |
| GO:0005198 | 5.37E-05 | 2.77E-03 | structural molecule activity |
| GO:0016459 | 6.86E-05 | 3.35E-03 | myosin complex |
| GO:0005815 | 6.89E-05 | 3.35E-03 | microtubule organizing center |
| GO:0004521 | 9.09E-05 | 4.20E-03 | endoribonuclease activity |
| GO:0006412 | 9.12E-05 | 4.20E-03 | translation |
| GO:0016891 | 1.28E-04 | 5.72E-03 | endoribonuclease activity, producing 5'-phosphomonoesters |
| GO:0004523 | 1.35E-04 | 5.90E-03 | ribonuclease H activity |
| GO:0004540 | 1.50E-04 | 6.38E-03 | ribonuclease activity |
| GO:0008270 | 1.84E-04 | 7.63E-03 | zinc ion binding |
| GO:0016893 | 2.13E-04 | 8.65E-03 | endonuclease activity, active with either ribo- or deoxyribonucleic acids and producing 5'-phosphomonoesters |
| GO:0043228 | 2.61E-04 | 1.01E-02 | non-membrane-bounded organelle |
| GO:0043232 | 2.61E-04 | 1.01E-02 | intracellular non-membrane-bounded organelle |
| GO:0032982 | 3.79E-04 | 1.43E-02 | myosin filament |
| GO:0008152 | 5.69E-04 | 2.11E-02 | metabolic process |
| GO:0016812 | 6.21E-04 | 2.25E-02 | hydrolase activity, acting on carbon-nitrogen (but not peptide) bonds, in cyclic amides |
| GO:0003684 | 8.10E-04 | 2.88E-02 | damaged DNA binding |
| GO:0009452 | 1.23E-03 | 4.29E-02 | RNA capping |
| GO:0033554 | 1.49E-03 | 4.77E-02 | cellular response to stress |
| GO:0004482 | 1.51E-03 | 4.77E-02 | mRNA (guanine-N7-)-methyltransferase activity |
| GO:0004151 | 1.51E-03 | 4.77E-02 | dihydroorotase activity |
| GO:0050598 | 1.51E-03 | 4.77E-02 | taxane 13-alpha-hydroxylase activity |
| GO:0006995 | 1.51E-03 | 4.77E-02 | cellular response to nitrogen starvation |
| **1st leaf** |  |  |  |
| NA | NA | NA | NA |
| **2nd leaf** |  |  |  |
| GO-ID | p-value | corr p-value | Description |
| GO:0043234 | 3.74E-14 | 1.82E-10 | protein complex |
| GO:0044464 | 1.83E-13 | 2.96E-10 | cell part |
| GO:0005623 | 1.83E-13 | 2.96E-10 | cell |
| GO:0006886 | 9.72E-13 | 1.18E-09 | intracellular protein transport |
| GO:0051649 | 1.64E-12 | 1.54E-09 | establishment of localization in cell |
| GO:0046907 | 2.44E-12 | 1.54E-09 | intracellular transport |
| GO:0015031 | 2.53E-12 | 1.54E-09 | protein transport |
| GO:0045184 | 2.53E-12 | 1.54E-09 | establishment of protein localization |
| GO:0008104 | 3.70E-12 | 2.00E-09 | protein localization |
| GO:0005524 | 6.70E-12 | 3.26E-09 | ATP binding |
| GO:0034613 | 2.74E-11 | 1.21E-08 | cellular protein localization |
| GO:0051641 | 5.30E-11 | 2.15E-08 | cellular localization |
| GO:0005575 | 7.07E-11 | 2.64E-08 | cellular_component |
| GO:0070727 | 2.72E-10 | 9.37E-08 | cellular macromolecule localization |
| GO:0005634 | 2.89E-10 | 9.37E-08 | nucleus |
| GO:0050789 | 3.12E-10 | 9.47E-08 | regulation of biological process |
| GO:0016874 | 6.29E-10 | 1.80E-07 | ligase activity |
| GO:0003777 | 1.53E-09 | 4.13E-07 | microtubule motor activity |
| GO:0008287 | 5.42E-09 | 1.39E-06 | protein serine/threonine phosphatase complex |
| GO:0003774 | 1.04E-08 | 2.52E-06 | motor activity |
| GO:0004721 | 1.18E-08 | 2.74E-06 | phosphoprotein phosphatase activity |
| GO:0017111 | 1.46E-08 | 3.08E-06 | nucleoside-triphosphatase activity |
| GO:0050794 | 1.46E-08 | 3.08E-06 | regulation of cellular process |
| GO:0016791 | 4.07E-08 | 8.24E-06 | phosphatase activity |
| GO:0016817 | 5.90E-08 | 1.15E-05 | hydrolase activity, acting on acid anhydrides |
| GO:0016462 | 7.55E-08 | 1.41E-05 | pyrophosphatase activity |
| GO:0065007 | 9.98E-08 | 1.80E-05 | biological regulation |
| GO:0016192 | 1.17E-07 | 2.03E-05 | vesicle-mediated transport |
| GO:0023052 | 1.34E-07 | 2.24E-05 | signaling |
| GO:0030163 | 1.44E-07 | 2.26E-05 | protein catabolic process |
| GO:0044265 | 1.47E-07 | 2.26E-05 | cellular macromolecule catabolic process |
| GO:0005886 | 1.49E-07 | 2.26E-05 | plasma membrane |
| GO:0000166 | 1.60E-07 | 2.36E-05 | nucleotide binding |
| GO:0051603 | 1.66E-07 | 2.37E-05 | proteolysis involved in cellular protein catabolic process |
| GO:0043632 | 2.00E-07 | 2.61E-05 | modification-dependent macromolecule catabolic process |
| GO:0019941 | 2.00E-07 | 2.61E-05 | modification-dependent protein catabolic process |
| GO:0006511 | 2.00E-07 | 2.61E-05 | ubiquitin-dependent protein catabolic process |
| GO:0006396 | 2.04E-07 | 2.61E-05 | RNA processing |
| GO:0044257 | 2.14E-07 | 2.64E-05 | cellular protein catabolic process |
| GO:0009894 | 2.18E-07 | 2.64E-05 | regulation of catabolic process |
| GO:0033036 | 2.45E-07 | 2.91E-05 | macromolecule localization |
| GO:0005622 | 2.91E-07 | 3.37E-05 | intracellular |
| GO:0016818 | 3.00E-07 | 3.39E-05 | hydrolase activity, acting on acid anhydrides, in phosphorus-containing anhydrides |
| GO:0006470 | 3.22E-07 | 3.56E-05 | protein amino acid dephosphorylation |
| GO:0016568 | 3.52E-07 | 3.80E-05 | chromatin modification |
| GO:0001883 | 3.62E-07 | 3.82E-05 | purine nucleoside binding |
| GO:0004722 | 4.03E-07 | 4.13E-05 | protein serine/threonine phosphatase activity |
| GO:0016311 | 4.07E-07 | 4.13E-05 | dephosphorylation |
| GO:0043412 | 4.17E-07 | 4.14E-05 | macromolecule modification |
| GO:0030554 | 4.42E-07 | 4.22E-05 | adenyl nucleotide binding |
| GO:0060589 | 4.43E-07 | 4.22E-05 | nucleoside-triphosphatase regulator activity |
| GO:0006464 | 5.98E-07 | 5.59E-05 | protein modification process |
| GO:0000502 | 7.08E-07 | 6.49E-05 | proteasome complex |
| GO:0048580 | 7.72E-07 | 6.95E-05 | regulation of post-embryonic development |
| GO:0032559 | 9.02E-07 | 7.97E-05 | adenyl ribonucleotide binding |
| GO:0016570 | 1.02E-06 | 8.84E-05 | histone modification |
| GO:0006996 | 1.11E-06 | 9.46E-05 | organelle organization |
| GO:0030695 | 1.34E-06 | 1.11E-04 | GTPase regulator activity |
| GO:0043687 | 1.35E-06 | 1.11E-04 | post-translational protein modification |
| GO:0019222 | 1.46E-06 | 1.18E-04 | regulation of metabolic process |
| GO:0008565 | 1.64E-06 | 1.31E-04 | protein transporter activity |
| GO:0001882 | 1.70E-06 | 1.33E-04 | nucleoside binding |
| GO:0017076 | 1.92E-06 | 1.48E-04 | purine nucleotide binding |
| GO:0016070 | 2.45E-06 | 1.80E-04 | RNA metabolic process |
| GO:0004386 | 2.45E-06 | 1.80E-04 | helicase activity |
| GO:0035466 | 2.47E-06 | 1.80E-04 | regulation of signaling pathway |
| GO:0006457 | 2.48E-06 | 1.80E-04 | protein folding |
| GO:0031072 | 2.72E-06 | 1.94E-04 | heat shock protein binding |
| GO:0031329 | 2.98E-06 | 2.10E-04 | regulation of cellular catabolic process |
| GO:0042578 | 3.45E-06 | 2.39E-04 | phosphoric ester hydrolase activity |
| GO:0032555 | 4.11E-06 | 2.78E-04 | purine ribonucleotide binding |
| GO:0032553 | 4.11E-06 | 2.78E-04 | ribonucleotide binding |
| GO:0031323 | 5.50E-06 | 3.66E-04 | regulation of cellular metabolic process |
| GO:0016569 | 6.84E-06 | 4.49E-04 | covalent chromatin modification |
| GO:0004715 | 8.90E-06 | 5.77E-04 | non-membrane spanning protein tyrosine kinase activity |
| GO:0004221 | 1.21E-05 | 7.76E-04 | ubiquitin thiolesterase activity |
| GO:0051171 | 1.53E-05 | 9.64E-04 | regulation of nitrogen compound metabolic process |
| GO:0008237 | 1.83E-05 | 1.14E-03 | metallopeptidase activity |
| GO:0016627 | 1.91E-05 | 1.17E-03 | oxidoreductase activity, acting on the CH-CH group of donors |
| GO:0080090 | 2.13E-05 | 1.25E-03 | regulation of primary metabolic process |
| GO:0009725 | 2.13E-05 | 1.25E-03 | response to hormone stimulus |
| GO:0051056 | 2.14E-05 | 1.25E-03 | regulation of small GTPase mediated signal transduction |
| GO:0046578 | 2.14E-05 | 1.25E-03 | regulation of Ras protein signal transduction |
| GO:0033121 | 2.27E-05 | 1.30E-03 | regulation of purine nucleotide catabolic process |
| GO:0030811 | 2.27E-05 | 1.30E-03 | regulation of nucleotide catabolic process |
| GO:0043085 | 2.60E-05 | 1.47E-03 | positive regulation of catalytic activity |
| GO:0023033 | 2.81E-05 | 1.57E-03 | signaling pathway |
| GO:0010646 | 2.90E-05 | 1.60E-03 | regulation of cell communication |
| GO:0070647 | 3.07E-05 | 1.68E-03 | protein modification by small protein conjugation or removal |
| GO:0016585 | 3.21E-05 | 1.73E-03 | chromatin remodeling complex |
| GO:0000911 | 3.43E-05 | 1.82E-03 | cytokinesis by cell plate formation |
| GO:0005096 | 3.45E-05 | 1.82E-03 | GTPase activator activity |
| GO:0030117 | 4.04E-05 | 2.07E-03 | membrane coat |
| GO:0048475 | 4.04E-05 | 2.07E-03 | coated membrane |
| GO:0007165 | 4.05E-05 | 2.07E-03 | signal transduction |
| GO:0023060 | 4.21E-05 | 2.11E-03 | signal transmission |
| GO:0023046 | 4.21E-05 | 2.11E-03 | signaling process |
| GO:0016881 | 4.47E-05 | 2.22E-03 | acid-amino acid ligase activity |
| GO:0043087 | 4.93E-05 | 2.40E-03 | regulation of GTPase activity |
| GO:0033124 | 4.93E-05 | 2.40E-03 | regulation of GTP catabolic process |
| GO:0044093 | 5.40E-05 | 2.60E-03 | positive regulation of molecular function |
| GO:0009787 | 6.50E-05 | 3.10E-03 | regulation of abscisic acid mediated signaling pathway |
| GO:0043039 | 6.83E-05 | 3.19E-03 | tRNA aminoacylation |
| GO:0043038 | 6.83E-05 | 3.19E-03 | amino acid activation |
| GO:0051301 | 6.90E-05 | 3.19E-03 | cell division |
| GO:0005083 | 7.44E-05 | 3.41E-03 | small GTPase regulator activity |
| GO:0019538 | 7.56E-05 | 3.43E-03 | protein metabolic process |
| GO:0005794 | 7.78E-05 | 3.50E-03 | Golgi apparatus |
| GO:0034660 | 8.18E-05 | 3.65E-03 | ncRNA metabolic process |
| GO:0019219 | 8.46E-05 | 3.74E-03 | regulation of nucleobase, nucleoside, nucleotide and nucleic acid metabolic process |
| GO:0009057 | 8.53E-05 | 3.74E-03 | macromolecule catabolic process |
| GO:0043565 | 8.68E-05 | 3.77E-03 | sequence-specific DNA binding |
| GO:0006140 | 8.88E-05 | 3.82E-03 | regulation of nucleotide metabolic process |
| GO:0016571 | 9.26E-05 | 3.95E-03 | histone methylation |
| GO:0006418 | 9.95E-05 | 4.19E-03 | tRNA aminoacylation for protein translation |
| GO:0016879 | 1.00E-04 | 4.19E-03 | ligase activity, forming carbon-nitrogen bonds |
| GO:0000151 | 1.02E-04 | 4.23E-03 | ubiquitin ligase complex |
| GO:0008047 | 1.03E-04 | 4.26E-03 | enzyme activator activity |
| GO:0016875 | 1.08E-04 | 4.33E-03 | ligase activity, forming carbon-oxygen bonds |
| GO:0016876 | 1.08E-04 | 4.33E-03 | ligase activity, forming aminoacyl-tRNA and related compounds |
| GO:0004812 | 1.08E-04 | 4.33E-03 | aminoacyl-tRNA ligase activity |
| GO:0044267 | 1.10E-04 | 4.38E-03 | cellular protein metabolic process |
| GO:0005085 | 1.17E-04 | 4.60E-03 | guanyl-nucleotide exchange factor activity |
| GO:0008213 | 1.20E-04 | 4.68E-03 | protein amino acid alkylation |
| GO:0006479 | 1.20E-04 | 4.68E-03 | protein amino acid methylation |
| GO:0006325 | 1.30E-04 | 5.00E-03 | chromatin organization |
| GO:0009719 | 1.32E-04 | 5.06E-03 | response to endogenous stimulus |
| GO:0043547 | 1.35E-04 | 5.13E-03 | positive regulation of GTPase activity |
| GO:0004683 | 1.50E-04 | 5.63E-03 | calmodulin-dependent protein kinase activity |
| GO:0051082 | 1.60E-04 | 6.00E-03 | unfolded protein binding |
| GO:0031461 | 1.65E-04 | 6.11E-03 | cullin-RING ubiquitin ligase complex |
| GO:0051276 | 1.70E-04 | 6.26E-03 | chromosome organization |
| GO:0009909 | 1.74E-04 | 6.36E-03 | regulation of flower development |
| GO:0044428 | 1.87E-04 | 6.78E-03 | nuclear part |
| GO:0033205 | 1.92E-04 | 6.90E-03 | cell cycle cytokinesis |
| GO:0030120 | 1.96E-04 | 7.01E-03 | vesicle coat |
| GO:0008380 | 2.03E-04 | 7.21E-03 | RNA splicing |
| GO:0016790 | 2.44E-04 | 8.59E-03 | thiolester hydrolase activity |
| GO:0016567 | 2.65E-04 | 9.24E-03 | protein ubiquitination |
| GO:0032446 | 2.66E-04 | 9.24E-03 | protein modification by small protein conjugation |
| GO:0006399 | 2.70E-04 | 9.31E-03 | tRNA metabolic process |
| GO:0019787 | 2.92E-04 | 1.00E-02 | small conjugating protein ligase activity |
| GO:0009889 | 3.03E-04 | 1.03E-02 | regulation of biosynthetic process |
| GO:0003677 | 3.08E-04 | 1.04E-02 | DNA binding |
| GO:0004842 | 3.45E-04 | 1.15E-02 | ubiquitin-protein ligase activity |
| GO:0060255 | 3.48E-04 | 1.16E-02 | regulation of macromolecule metabolic process |
| GO:0000910 | 3.75E-04 | 1.24E-02 | cytokinesis |
| GO:0016634 | 3.88E-04 | 1.27E-02 | oxidoreductase activity, acting on the CH-CH group of donors, oxygen as acceptor |
| GO:0010468 | 4.01E-04 | 1.31E-02 | regulation of gene expression |
| GO:0030662 | 4.07E-04 | 1.32E-02 | coated vesicle membrane |
| GO:0031326 | 4.16E-04 | 1.34E-02 | regulation of cellular biosynthetic process |
| GO:0050793 | 4.20E-04 | 1.34E-02 | regulation of developmental process |
| GO:0009574 | 4.56E-04 | 1.45E-02 | preprophase band |
| GO:0006352 | 5.06E-04 | 1.60E-02 | transcription initiation |
| GO:0016773 | 5.71E-04 | 1.79E-02 | phosphotransferase activity, alcohol group as acceptor |
| GO:0005034 | 5.74E-04 | 1.79E-02 | osmosensor activity |
| GO:0032318 | 5.81E-04 | 1.80E-02 | regulation of Ras GTPase activity |
| GO:0019888 | 5.89E-04 | 1.80E-02 | protein phosphatase regulator activity |
| GO:0019208 | 5.89E-04 | 1.80E-02 | phosphatase regulator activity |
| GO:0051239 | 6.07E-04 | 1.85E-02 | regulation of multicellular organismal process |
| GO:0016485 | 6.17E-04 | 1.86E-02 | protein processing |
| GO:0005635 | 7.06E-04 | 2.12E-02 | nuclear envelope |
| GO:0006888 | 7.29E-04 | 2.17E-02 | ER to Golgi vesicle-mediated transport |
| GO:0005543 | 7.35E-04 | 2.18E-02 | phospholipid binding |
| GO:0042623 | 7.41E-04 | 2.18E-02 | ATPase activity, coupled |
| GO:0051170 | 8.65E-04 | 2.52E-02 | nuclear import |
| GO:0006270 | 8.65E-04 | 2.52E-02 | DNA-dependent DNA replication initiation |
| GO:0003743 | 8.82E-04 | 2.54E-02 | translation initiation factor activity |
| GO:0034968 | 8.84E-04 | 2.54E-02 | histone lysine methylation |
| GO:0043543 | 9.05E-04 | 2.57E-02 | protein amino acid acylation |
| GO:0008235 | 9.05E-04 | 2.57E-02 | metalloexopeptidase activity |
| GO:0006605 | 9.58E-04 | 2.71E-02 | protein targeting |
| GO:0044433 | 9.73E-04 | 2.72E-02 | cytoplasmic vesicle part |
| GO:0030659 | 9.73E-04 | 2.72E-02 | cytoplasmic vesicle membrane |
| GO:0048193 | 1.00E-03 | 2.78E-02 | Golgi vesicle transport |
| GO:0032440 | 1.05E-03 | 2.90E-02 | 2-alkenal reductase activity |
| GO:0090056 | 1.08E-03 | 2.96E-02 | regulation of chlorophyll metabolic process |
| GO:0051193 | 1.08E-03 | 2.96E-02 | regulation of cofactor metabolic process |
| GO:0051345 | 1.09E-03 | 2.96E-02 | positive regulation of hydrolase activity |
| GO:0010556 | 1.10E-03 | 2.96E-02 | regulation of macromolecule biosynthetic process |
| GO:0000159 | 1.12E-03 | 3.01E-02 | protein phosphatase type 2A complex |
| GO:0031968 | 1.15E-03 | 3.08E-02 | organelle outer membrane |
| GO:0045449 | 1.18E-03 | 3.09E-02 | regulation of transcription |
| GO:0004822 | 1.18E-03 | 3.09E-02 | isoleucine-tRNA ligase activity |
| GO:0000226 | 1.18E-03 | 3.09E-02 | microtubule cytoskeleton organization |
| GO:0000338 | 1.19E-03 | 3.09E-02 | protein deneddylation |
| GO:0010388 | 1.19E-03 | 3.09E-02 | cullin deneddylation |
| GO:0008276 | 1.20E-03 | 3.10E-02 | protein methyltransferase activity |
| GO:0070035 | 1.23E-03 | 3.14E-02 | purine NTP-dependent helicase activity |
| GO:0008026 | 1.23E-03 | 3.14E-02 | ATP-dependent helicase activity |
| GO:0051252 | 1.32E-03 | 3.35E-02 | regulation of RNA metabolic process |
| GO:0009658 | 1.34E-03 | 3.40E-02 | chloroplast organization |
| GO:0051640 | 1.36E-03 | 3.41E-02 | organelle localization |
| GO:0005643 | 1.36E-03 | 3.41E-02 | nuclear pore |
| GO:0006355 | 1.45E-03 | 3.62E-02 | regulation of transcription, DNA-dependent |
| GO:0009966 | 1.58E-03 | 3.90E-02 | regulation of signal transduction |
| GO:0023051 | 1.58E-03 | 3.90E-02 | regulation of signaling process |
| GO:0016810 | 1.67E-03 | 4.10E-02 | hydrolase activity, acting on carbon-nitrogen (but not peptide) bonds |
| GO:0016071 | 1.71E-03 | 4.16E-02 | mRNA metabolic process |
| GO:0006606 | 1.71E-03 | 4.16E-02 | protein import into nucleus |
| GO:0000156 | 1.76E-03 | 4.25E-02 | two-component response regulator activity |
| GO:0071495 | 1.78E-03 | 4.29E-02 | cellular response to endogenous stimulus |
| GO:0070646 | 1.93E-03 | 4.52E-02 | protein modification by small protein removal |
| GO:0051656 | 1.93E-03 | 4.52E-02 | establishment of organelle localization |
| GO:0000160 | 1.94E-03 | 4.52E-02 | two-component signal transduction system (phosphorelay) |
| GO:0051604 | 1.96E-03 | 4.52E-02 | protein maturation |
| GO:0032320 | 1.97E-03 | 4.52E-02 | positive regulation of Ras GTPase activity |
| GO:0032012 | 1.97E-03 | 4.52E-02 | regulation of ARF protein signal transduction |
| GO:0032147 | 1.98E-03 | 4.52E-02 | activation of protein kinase activity |
| GO:0004160 | 1.99E-03 | 4.52E-02 | dihydroxy-acid dehydratase activity |
| GO:0046785 | 1.99E-03 | 4.52E-02 | microtubule polymerization |
| GO:0031109 | 1.99E-03 | 4.52E-02 | microtubule polymerization or depolymerization |
| GO:0015415 | 1.99E-03 | 4.52E-02 | phosphate transmembrane-transporting ATPase activity |
| GO:0033365 | 1.99E-03 | 4.52E-02 | protein localization in organelle |
| GO:0032259 | 2.00E-03 | 4.52E-02 | methylation |
| GO:0032870 | 2.04E-03 | 4.59E-02 | cellular response to hormone stimulus |
| GO:0008601 | 2.08E-03 | 4.66E-02 | protein phosphatase type 2A regulator activity |
| GO:0016573 | 2.12E-03 | 4.73E-02 | histone acetylation |
| GO:0008194 | 2.13E-03 | 4.74E-02 | UDP-glycosyltransferase activity |
| GO:0048585 | 2.14E-03 | 4.74E-02 | negative regulation of response to stimulus |
| **Mature leaf** | |  |  |
| GO:0003964 | 1.09E-07 | 3.39E-05 | RNA-directed DNA polymerase activity |
| GO:0006278 | 1.38E-07 | 3.39E-05 | RNA-dependent DNA replication |
| GO:0034061 | 1.85E-07 | 3.39E-05 | DNA polymerase activity |
| GO:0016772 | 1.87E-07 | 3.39E-05 | transferase activity, transferring phosphorus-containing groups |
| GO:0016779 | 4.85E-07 | 7.05E-05 | nucleotidyltransferase activity |
| GO:0004523 | 7.05E-07 | 8.53E-05 | ribonuclease H activity |
| GO:0006260 | 1.05E-06 | 1.08E-04 | DNA replication |
| GO:0016891 | 1.20E-06 | 1.08E-04 | endoribonuclease activity, producing 5'-phosphomonoesters |
| GO:0016893 | 1.64E-06 | 1.21E-04 | endonuclease activity, active with either ribo- or deoxyribonucleic acids and producing 5'-phosphomonoesters |
| GO:0004521 | 1.67E-06 | 1.21E-04 | endoribonuclease activity |
| GO:0004540 | 3.71E-06 | 2.32E-04 | ribonuclease activity |
| GO:0016740 | 3.83E-06 | 2.32E-04 | transferase activity |
| GO:0016788 | 6.12E-06 | 3.42E-04 | hydrolase activity, acting on ester bonds |
| GO:0003922 | 1.09E-05 | 5.64E-04 | GMP synthase (glutamine-hydrolyzing) activity |
| GO:0004519 | 1.17E-05 | 5.68E-04 | endonuclease activity |
| GO:0003824 | 6.82E-05 | 3.04E-03 | catalytic activity |
| GO:0004518 | 7.13E-05 | 3.04E-03 | nuclease activity |
| GO:0003723 | 9.57E-05 | 3.86E-03 | RNA binding |
| GO:0046037 | 1.08E-04 | 3.92E-03 | GMP metabolic process |
| GO:0006177 | 1.08E-04 | 3.92E-03 | GMP biosynthetic process |
| GO:0015385 | 1.97E-04 | 6.81E-03 | sodium:hydrogen antiporter activity |
| GO:0005451 | 2.54E-04 | 8.37E-03 | monovalent cation:hydrogen antiporter activity |
| GO:0006885 | 2.75E-04 | 8.68E-03 | regulation of pH |
| GO:0055067 | 3.45E-04 | 1.04E-02 | monovalent inorganic cation homeostasis |
| GO:0022804 | 3.67E-04 | 1.07E-02 | active transmembrane transporter activity |
| GO:0016787 | 3.93E-04 | 1.10E-02 | hydrolase activity |
| GO:0015491 | 4.25E-04 | 1.14E-02 | cation:cation antiporter activity |
| GO:0009052 | 9.64E-04 | 2.41E-02 | pentose-phosphate shunt, non-oxidative branch |
| GO:0004751 | 9.64E-04 | 2.41E-02 | ribose-5-phosphate isomerase activity |
| GO:0006468 | 1.20E-03 | 2.92E-02 | protein amino acid phosphorylation |
| GO:0004672 | 1.50E-03 | 3.52E-02 | protein kinase activity |
| GO:0006200 | 1.82E-03 | 4.14E-02 | ATP catabolic process |
| GO:0006259 | 2.19E-03 | 4.71E-02 | DNA metabolic process |
| GO:0006139 | 2.20E-03 | 4.71E-02 | nucleobase, nucleoside, nucleotide and nucleic acid metabolic process |
| GO:0045330 | 2.33E-03 | 4.84E-02 | aspartyl esterase activity |
| **Old leaf** |  |  |  |
| GO:0006259 | 2.27E-64 | 2.33E-61 | DNA metabolic process |
| GO:0003964 | 2.47E-46 | 1.27E-43 | RNA-directed DNA polymerase activity |
| GO:0034061 | 7.40E-46 | 2.54E-43 | DNA polymerase activity |
| GO:0016772 | 7.56E-45 | 1.94E-42 | transferase activity, transferring phosphorus-containing groups |
| GO:0006278 | 2.30E-44 | 4.72E-42 | RNA-dependent DNA replication |
| GO:0006260 | 4.28E-41 | 6.65E-39 | DNA replication |
| GO:0015074 | 4.53E-41 | 6.65E-39 | DNA integration |
| GO:0016779 | 6.15E-41 | 7.90E-39 | nucleotidyltransferase activity |
| GO:0090304 | 4.25E-38 | 4.86E-36 | nucleic acid metabolic process |
| GO:0044260 | 2.50E-37 | 2.57E-35 | cellular macromolecule metabolic process |
| GO:0043170 | 2.01E-36 | 1.88E-34 | macromolecule metabolic process |
| GO:0006139 | 6.80E-31 | 5.82E-29 | nucleobase, nucleoside, nucleotide and nucleic acid metabolic process |
| GO:0003723 | 2.39E-27 | 1.89E-25 | RNA binding |
| GO:0003676 | 3.26E-26 | 2.39E-24 | nucleic acid binding |
| GO:0016740 | 5.42E-25 | 3.72E-23 | transferase activity |
| GO:0034641 | 1.03E-23 | 6.65E-22 | cellular nitrogen compound metabolic process |
| GO:0006807 | 3.35E-23 | 2.02E-21 | nitrogen compound metabolic process |
| GO:0008037 | 3.04E-19 | 1.65E-17 | cell recognition |
| GO:0048544 | 3.04E-19 | 1.65E-17 | recognition of pollen |
| GO:0009875 | 6.20E-19 | 3.19E-17 | pollen-pistil interaction |
| GO:0044238 | 1.35E-18 | 6.63E-17 | primary metabolic process |
| GO:0044237 | 3.30E-18 | 1.54E-16 | cellular metabolic process |
| GO:0004521 | 2.44E-16 | 1.09E-14 | endoribonuclease activity |
| GO:0016891 | 5.56E-16 | 2.38E-14 | endoribonuclease activity, producing 5'-phosphomonoesters |
| GO:0004519 | 7.07E-16 | 2.91E-14 | endonuclease activity |
| GO:0004523 | 7.42E-16 | 2.93E-14 | ribonuclease H activity |
| GO:0016893 | 1.46E-15 | 5.58E-14 | endonuclease activity, active with either ribo- or deoxyribonucleic acids and producing 5'-phosphomonoesters |
| GO:0004540 | 3.04E-15 | 1.11E-13 | ribonuclease activity |
| GO:0009856 | 2.53E-14 | 8.96E-13 | pollination |
| GO:0048610 | 2.97E-14 | 1.02E-12 | reproductive cellular process |
| GO:0007154 | 3.97E-14 | 1.32E-12 | cell communication |
| GO:0034645 | 2.50E-13 | 8.03E-12 | cellular macromolecule biosynthetic process |
| GO:0009059 | 2.76E-13 | 8.59E-12 | macromolecule biosynthetic process |
| GO:0004518 | 3.59E-13 | 1.09E-11 | nuclease activity |
| GO:0006468 | 4.21E-13 | 1.24E-11 | protein amino acid phosphorylation |
| GO:0004672 | 1.08E-12 | 3.07E-11 | protein kinase activity |
| GO:0005488 | 9.08E-12 | 2.52E-10 | binding |
| GO:0008152 | 2.11E-11 | 5.71E-10 | metabolic process |
| GO:0016773 | 8.79E-11 | 2.32E-09 | phosphotransferase activity, alcohol group as acceptor |
| GO:0043687 | 1.47E-10 | 3.79E-09 | post-translational protein modification |
| GO:0009987 | 2.37E-10 | 5.94E-09 | cellular process |
| GO:0004674 | 5.06E-10 | 1.24E-08 | protein serine/threonine kinase activity |
| GO:0008270 | 5.29E-10 | 1.26E-08 | zinc ion binding |
| GO:0016310 | 7.78E-10 | 1.82E-08 | phosphorylation |
| GO:0016301 | 1.26E-09 | 2.88E-08 | kinase activity |
| GO:0006464 | 4.31E-09 | 9.64E-08 | protein modification process |
| GO:0004713 | 5.36E-09 | 1.17E-07 | protein tyrosine kinase activity |
| GO:0006796 | 9.20E-09 | 1.97E-07 | phosphate metabolic process |
| GO:0006793 | 9.53E-09 | 2.00E-07 | phosphorus metabolic process |
| GO:0051704 | 1.19E-08 | 2.45E-07 | multi-organism process |
| GO:0003674 | 2.15E-08 | 4.34E-07 | molecular_function |
| GO:0043412 | 4.03E-08 | 7.97E-07 | macromolecule modification |
| GO:0003824 | 1.61E-07 | 3.13E-06 | catalytic activity |
| GO:0016788 | 8.60E-07 | 1.64E-05 | hydrolase activity, acting on ester bonds |
| GO:0002764 | 3.69E-06 | 6.90E-05 | immune response-regulating signaling pathway |
| GO:0030554 | 4.11E-06 | 7.52E-05 | adenyl nucleotide binding |
| GO:0001883 | 4.17E-06 | 7.52E-05 | purine nucleoside binding |
| GO:0044249 | 4.56E-06 | 8.08E-05 | cellular biosynthetic process |
| GO:0016045 | 5.75E-06 | 1.00E-04 | detection of bacterium |
| GO:0032559 | 7.08E-06 | 1.21E-04 | adenyl ribonucleotide binding |
| GO:0022414 | 7.41E-06 | 1.25E-04 | reproductive process |
| GO:0001882 | 7.73E-06 | 1.28E-04 | nucleoside binding |
| GO:0004190 | 8.28E-06 | 1.33E-04 | aspartic-type endopeptidase activity |
| GO:0070001 | 8.28E-06 | 1.33E-04 | aspartic-type peptidase activity |
| GO:0000003 | 1.02E-05 | 1.62E-04 | reproduction |
| GO:0010359 | 1.70E-05 | 2.60E-04 | regulation of anion channel activity |
| GO:0009595 | 1.70E-05 | 2.60E-04 | detection of biotic stimulus |
| GO:0009058 | 2.45E-05 | 3.70E-04 | biosynthetic process |
| GO:0010204 | 3.03E-05 | 4.51E-04 | defense response signaling pathway, resistance gene-independent |
| GO:0032409 | 3.92E-05 | 5.60E-04 | regulation of transporter activity |
| GO:0032412 | 3.92E-05 | 5.60E-04 | regulation of ion transmembrane transporter activity |
| GO:0022898 | 3.92E-05 | 5.60E-04 | regulation of transmembrane transporter activity |
| GO:0005524 | 4.10E-05 | 5.78E-04 | ATP binding |
| GO:0046914 | 7.54E-05 | 1.05E-03 | transition metal ion binding |
| GO:0019538 | 9.91E-05 | 1.36E-03 | protein metabolic process |
| GO:0034762 | 1.15E-04 | 1.54E-03 | regulation of transmembrane transport |
| GO:0034765 | 1.15E-04 | 1.54E-03 | regulation of ion transmembrane transport |
| GO:0002218 | 2.26E-04 | 2.95E-03 | activation of innate immune response |
| GO:0002253 | 2.26E-04 | 2.95E-03 | activation of immune response |
| GO:0045089 | 4.00E-04 | 5.02E-03 | positive regulation of innate immune response |
| GO:0002684 | 4.00E-04 | 5.02E-03 | positive regulation of immune system process |
| GO:0050778 | 4.00E-04 | 5.02E-03 | positive regulation of immune response |
| GO:0017076 | 4.10E-04 | 5.08E-03 | purine nucleotide binding |
| GO:0034050 | 4.55E-04 | 5.50E-03 | host programmed cell death induced by symbiont |
| GO:0009626 | 4.55E-04 | 5.50E-03 | plant-type hypersensitive response |
| GO:0031349 | 6.52E-04 | 7.80E-03 | positive regulation of defense response |
| GO:0032555 | 6.68E-04 | 7.81E-03 | purine ribonucleotide binding |
| GO:0032553 | 6.68E-04 | 7.81E-03 | ribonucleotide binding |
| GO:0045088 | 8.14E-04 | 9.40E-03 | regulation of innate immune response |
| GO:0008150 | 1.09E-03 | 1.25E-02 | biological_process |
| GO:0032147 | 1.40E-03 | 1.58E-02 | activation of protein kinase activity |
| GO:0045860 | 1.49E-03 | 1.65E-02 | positive regulation of protein kinase activity |
| GO:0033674 | 1.49E-03 | 1.65E-02 | positive regulation of kinase activity |
| GO:0050776 | 1.60E-03 | 1.75E-02 | regulation of immune response |
| GO:0051347 | 1.69E-03 | 1.83E-02 | positive regulation of transferase activity |
| GO:0002682 | 1.74E-03 | 1.87E-02 | regulation of immune system process |
| GO:0000186 | 1.89E-03 | 2.01E-02 | activation of MAPKK activity |
| GO:0004709 | 2.06E-03 | 2.16E-02 | MAP kinase kinase kinase activity |
| GO:0004175 | 2.21E-03 | 2.29E-02 | endopeptidase activity |
| GO:0044070 | 2.23E-03 | 2.29E-02 | regulation of anion transport |
| GO:0044267 | 2.31E-03 | 2.35E-02 | cellular protein metabolic process |
| GO:0005452 | 3.03E-03 | 3.03E-02 | inorganic anion exchanger activity |
| GO:0015380 | 3.03E-03 | 3.03E-02 | anion exchanger activity |
| GO:0048584 | 3.96E-03 | 3.91E-02 | positive regulation of response to stimulus |
| GO:0015301 | 4.21E-03 | 4.09E-02 | anion:anion antiporter activity |
| GO:0031347 | 4.22E-03 | 4.09E-02 | regulation of defense response |
| **Stem** |  |  |  |
| GO:0003735 | 2.69E-05 | 1.06E-02 | structural constituent of ribosome |
| GO:0034645 | 2.72E-05 | 1.06E-02 | cellular macromolecule biosynthetic process |
| GO:0031012 | 2.85E-05 | 1.06E-02 | extracellular matrix |
| GO:0009059 | 2.86E-05 | 1.06E-02 | macromolecule biosynthetic process |
| GO:0006412 | 3.49E-05 | 1.06E-02 | translation |
| GO:0005198 | 4.95E-05 | 1.10E-02 | structural molecule activity |
| GO:0006260 | 5.08E-05 | 1.10E-02 | DNA replication |
| GO:0006278 | 8.33E-05 | 1.49E-02 | RNA-dependent DNA replication |
| GO:0030529 | 9.36E-05 | 1.49E-02 | ribonucleoprotein complex |
| GO:0044421 | 1.06E-04 | 1.49E-02 | extracellular region part |
| GO:0071229 | 1.08E-04 | 1.49E-02 | cellular response to acid |
| GO:0003723 | 1.24E-04 | 1.49E-02 | RNA binding |
| GO:0005840 | 1.28E-04 | 1.49E-02 | ribosome |
| GO:0003964 | 1.59E-04 | 1.72E-02 | RNA-directed DNA polymerase activity |
| GO:0033279 | 2.62E-04 | 2.28E-02 | ribosomal subunit |
| GO:0034061 | 2.87E-04 | 2.28E-02 | DNA polymerase activity |
| GO:0030198 | 2.97E-04 | 2.28E-02 | extracellular matrix organization |
| GO:0051098 | 2.97E-04 | 2.28E-02 | regulation of binding |
| GO:0051099 | 3.22E-04 | 2.28E-02 | positive regulation of binding |
| GO:0016708 | 3.22E-04 | 2.28E-02 | oxidoreductase activity, acting on paired donors, with incorporation or reduction of molecular oxygen, NADH or NADPH as one donor, and incorporation of two atoms of oxygen into one donor |
| GO:0016779 | 3.29E-04 | 2.28E-02 | nucleotidyltransferase activity |
| GO:0005615 | 3.31E-04 | 2.28E-02 | extracellular space |
| GO:0043062 | 3.74E-04 | 2.47E-02 | extracellular structure organization |
| GO:0016528 | 6.40E-04 | 3.88E-02 | sarcoplasm |
| GO:0016529 | 6.40E-04 | 3.88E-02 | sarcoplasmic reticulum |
| GO:0005578 | 6.83E-04 | 3.99E-02 | proteinaceous extracellular matrix |
| GO:0044249 | 7.78E-04 | 4.37E-02 | cellular biosynthetic process |
| **Flower** |  |  |  |
| GO:0005576 | 4.07E-06 | 5.95E-03 | extracellular region |
| GO:0004650 | 2.38E-05 | 1.74E-02 | polygalacturonase activity |
| GO:0044421 | 5.69E-05 | 2.78E-02 | extracellular region part |
| GO:0022857 | 1.43E-04 | 3.49E-02 | transmembrane transporter activity |
| GO:0015238 | 2.22E-04 | 3.49E-02 | drug transmembrane transporter activity |
| GO:0005615 | 2.26E-04 | 3.49E-02 | extracellular space |
| GO:0004482 | 2.64E-04 | 3.49E-02 | mRNA (guanine-N7-)-methyltransferase activity |
| GO:0006855 | 4.70E-04 | 3.49E-02 | drug transmembrane transport |
| GO:0015893 | 4.70E-04 | 3.49E-02 | drug transport |
| GO:0005578 | 5.11E-04 | 3.49E-02 | proteinaceous extracellular matrix |
| GO:0030027 | 5.24E-04 | 3.49E-02 | lamellipodium |
| GO:0010740 | 5.24E-04 | 3.49E-02 | positive regulation of intracellular protein kinase cascade |
| GO:0006903 | 5.24E-04 | 3.49E-02 | vesicle targeting |
| GO:0048858 | 5.24E-04 | 3.49E-02 | cell projection morphogenesis |
| GO:0009967 | 5.24E-04 | 3.49E-02 | positive regulation of signal transduction |
| GO:0090114 | 5.24E-04 | 3.49E-02 | COPII-coated vesicle budding |
| GO:0005344 | 5.24E-04 | 3.49E-02 | oxygen transporter activity |
| GO:0008174 | 5.24E-04 | 3.49E-02 | mRNA methyltransferase activity |
| GO:0048208 | 5.24E-04 | 3.49E-02 | COPII vesicle coating |
| GO:0048199 | 5.24E-04 | 3.49E-02 | vesicle targeting, to, from or within Golgi |
| GO:0048207 | 5.24E-04 | 3.49E-02 | vesicle targeting, rough ER to cis-Golgi |
| GO:0023056 | 5.24E-04 | 3.49E-02 | positive regulation of signaling process |
| GO:0042493 | 6.32E-04 | 4.02E-02 | response to drug |
| GO:0003735 | 8.43E-04 | 4.80E-02 | structural constituent of ribosome |
| GO:0010627 | 8.68E-04 | 4.80E-02 | regulation of intracellular protein kinase cascade |
| GO:0001101 | 8.68E-04 | 4.80E-02 | response to acid |
| GO:0022804 | 8.85E-04 | 4.80E-02 | active transmembrane transporter activity |
| **Seed** |  |  |  |
| GO:0006259 | 4.18E-35 | 4.53E-32 | DNA metabolic process |
| GO:0006278 | 1.54E-32 | 8.33E-30 | RNA-dependent DNA replication |
| GO:0003964 | 7.39E-32 | 2.67E-29 | RNA-directed DNA polymerase activity |
| GO:0034061 | 6.48E-31 | 1.75E-28 | DNA polymerase activity |
| GO:0006260 | 1.49E-29 | 3.21E-27 | DNA replication |
| GO:0090304 | 9.35E-29 | 1.69E-26 | nucleic acid metabolic process |
| GO:0016779 | 6.00E-28 | 9.28E-26 | nucleotidyltransferase activity |
| GO:0003723 | 4.89E-23 | 6.61E-21 | RNA binding |
| GO:0006139 | 4.57E-22 | 5.49E-20 | nucleobase, nucleoside, nucleotide and nucleic acid metabolic process |
| GO:0003676 | 1.45E-20 | 1.57E-18 | nucleic acid binding |
| GO:0034641 | 2.51E-18 | 2.47E-16 | cellular nitrogen compound metabolic process |
| GO:0006807 | 1.21E-17 | 1.09E-15 | nitrogen compound metabolic process |
| GO:0016772 | 7.93E-15 | 6.60E-13 | transferase activity, transferring phosphorus-containing groups |
| GO:0043170 | 9.12E-15 | 7.05E-13 | macromolecule metabolic process |
| GO:0034645 | 1.10E-12 | 7.94E-11 | cellular macromolecule biosynthetic process |
| GO:0009059 | 1.20E-12 | 8.14E-11 | macromolecule biosynthetic process |
| GO:0015074 | 1.30E-12 | 8.28E-11 | DNA integration |
| GO:0044260 | 4.63E-12 | 2.78E-10 | cellular macromolecule metabolic process |
| GO:0004523 | 7.75E-12 | 4.42E-10 | ribonuclease H activity |
| GO:0016891 | 2.58E-11 | 1.39E-09 | endoribonuclease activity, producing 5'-phosphomonoesters |
| GO:0016893 | 5.27E-11 | 2.70E-09 | endonuclease activity, active with either ribo- or deoxyribonucleic acids and producing 5'-phosphomonoesters |
| GO:0004521 | 5.49E-11 | 2.70E-09 | endoribonuclease activity |
| GO:0004519 | 2.06E-10 | 9.71E-09 | endonuclease activity |
| GO:0004540 | 3.31E-10 | 1.49E-08 | ribonuclease activity |
| GO:0016740 | 2.78E-09 | 1.20E-07 | transferase activity |
| GO:0044238 | 7.83E-09 | 3.26E-07 | primary metabolic process |
| GO:0004518 | 1.61E-08 | 6.44E-07 | nuclease activity |
| GO:0043531 | 1.13E-07 | 4.38E-06 | ADP binding |
| GO:0005488 | 4.66E-07 | 1.74E-05 | binding |
| GO:0006952 | 4.86E-07 | 1.75E-05 | defense response |
| GO:0044249 | 8.95E-07 | 3.12E-05 | cellular biosynthetic process |
| GO:0009058 | 2.60E-06 | 8.78E-05 | biosynthetic process |
| GO:0016788 | 5.22E-06 | 1.71E-04 | hydrolase activity, acting on ester bonds |
| GO:0043086 | 1.31E-05 | 4.17E-04 | negative regulation of catalytic activity |
| GO:0044092 | 1.65E-05 | 5.10E-04 | negative regulation of molecular function |
| GO:0050790 | 8.24E-05 | 2.48E-03 | regulation of catalytic activity |
| GO:0016787 | 8.82E-05 | 2.58E-03 | hydrolase activity |
| GO:0065009 | 1.41E-04 | 4.01E-03 | regulation of molecular function |
| GO:0044237 | 3.66E-04 | 1.02E-02 | cellular metabolic process |
| GO:0008889 | 8.90E-04 | 2.41E-02 | glycerophosphodiester phosphodiesterase activity |
| GO:0008233 | 1.05E-03 | 2.76E-02 | peptidase activity |
| GO:0006508 | 1.10E-03 | 2.83E-02 | proteolysis |
| GO:0004857 | 1.21E-03 | 3.06E-02 | enzyme inhibitor activity |
| GO:0006857 | 1.28E-03 | 3.12E-02 | oligopeptide transport |
| GO:0015833 | 1.34E-03 | 3.12E-02 | peptide transport |
| GO:0061135 | 1.35E-03 | 3.12E-02 | endopeptidase regulator activity |
| GO:0004866 | 1.35E-03 | 3.12E-02 | endopeptidase inhibitor activity |
| GO:0030414 | 1.43E-03 | 3.22E-02 | peptidase inhibitor activity |
| GO:0061134 | 1.68E-03 | 3.71E-02 | peptidase regulator activity |
| GO:0008150 | 1.92E-03 | 4.15E-02 | biological_process |
| GO:0004350 | 2.27E-03 | 4.81E-02 | glutamate-5-semialdehyde dehydrogenase activity |
| **Root** |  |  |  |
| GO:0020037 | 1.70E-12 | 1.99E-09 | heme binding |
| GO:0046906 | 7.79E-12 | 4.56E-09 | tetrapyrrole binding |
| GO:0005506 | 2.55E-11 | 9.96E-09 | iron ion binding |
| GO:0004601 | 2.07E-08 | 4.83E-06 | peroxidase activity |
| GO:0016684 | 2.07E-08 | 4.83E-06 | oxidoreductase activity, acting on peroxide as acceptor |
| GO:0016209 | 5.99E-08 | 1.17E-05 | antioxidant activity |
| GO:0016705 | 2.07E-07 | 3.46E-05 | oxidoreductase activity, acting on paired donors, with incorporation or reduction of molecular oxygen |
| GO:0047134 | 4.65E-07 | 6.80E-05 | protein-disulfide reductase activity |
| GO:0004497 | 8.06E-07 | 1.05E-04 | monooxygenase activity |
| GO:0010333 | 1.27E-06 | 1.49E-04 | terpene synthase activity |
| GO:0034002 | 2.03E-06 | 2.16E-04 | (R)-limonene synthase activity |
| GO:0015238 | 2.36E-06 | 2.30E-04 | drug transmembrane transporter activity |
| GO:0006855 | 3.25E-06 | 2.71E-04 | drug transmembrane transport |
| GO:0015893 | 3.25E-06 | 2.71E-04 | drug transport |
| GO:0042493 | 5.17E-06 | 4.03E-04 | response to drug |
| GO:0016838 | 6.93E-06 | 5.07E-04 | carbon-oxygen lyase activity, acting on phosphates |
| GO:0055085 | 1.31E-05 | 9.02E-04 | transmembrane transport |
| GO:0006979 | 2.05E-05 | 1.33E-03 | response to oxidative stress |
| GO:0016668 | 2.47E-05 | 1.52E-03 | oxidoreductase activity, acting on sulfur group of donors, NAD or NADP as acceptor |
| GO:0050550 | 5.01E-05 | 2.35E-03 | pinene synthase activity |
| GO:0050552 | 5.01E-05 | 2.35E-03 | (4S)-limonene synthase activity |
| GO:0080015 | 5.01E-05 | 2.35E-03 | sabinene synthase activity |
| GO:0016098 | 5.01E-05 | 2.35E-03 | monoterpenoid metabolic process |
| GO:0016099 | 5.01E-05 | 2.35E-03 | monoterpenoid biosynthetic process |
| GO:0034768 | 5.01E-05 | 2.35E-03 | (E)-beta-ocimene synthase activity |
| GO:0050551 | 7.95E-05 | 3.58E-03 | myrcene synthase activity |
| GO:0046914 | 1.19E-04 | 5.14E-03 | transition metal ion binding |
| GO:0080019 | 1.30E-04 | 5.36E-03 | fatty-acyl-CoA reductase (alcohol-forming) activity |
| GO:0004872 | 1.33E-04 | 5.36E-03 | receptor activity |
| GO:0055114 | 1.52E-04 | 5.92E-03 | oxidation reduction |
| GO:0043693 | 1.68E-04 | 6.12E-03 | monoterpene biosynthetic process |
| GO:0043692 | 1.68E-04 | 6.12E-03 | monoterpene metabolic process |
| GO:0009055 | 2.45E-04 | 8.69E-03 | electron carrier activity |
| GO:0016747 | 3.79E-04 | 1.30E-02 | transferase activity, transferring acyl groups other than amino-acyl groups |
| GO:0046872 | 4.00E-04 | 1.34E-02 | metal ion binding |
| GO:0004636 | 7.70E-04 | 2.50E-02 | phosphoribosyl-ATP diphosphatase activity |
| GO:0016491 | 7.92E-04 | 2.50E-02 | oxidoreductase activity |
| GO:0009975 | 1.25E-03 | 3.86E-02 | cyclase activity |
| GO:0009625 | 1.46E-03 | 4.39E-02 | response to insect |
| GO:0070838 | 1.51E-03 | 4.43E-02 | divalent metal ion transport |
| GO:0015297 | 1.55E-03 | 4.43E-02 | antiporter activity |
| GO:0009581 | 1.70E-03 | 4.73E-02 | detection of external stimulus |
